# The molecular logic of Nanog-induced self-renewal in mouse embryonic stem cells

**DOI:** 10.1038/s41467-019-09041-z

**Published:** 2019-03-07

**Authors:** Victor Heurtier, Nick Owens, Inma Gonzalez, Florian Mueller, Caroline Proux, Damien Mornico, Philippe Clerc, Agnes Dubois, Pablo Navarro

**Affiliations:** 1 Epigenetics of Stem Cells, Department of Developmental and Stem Cell Biology, Equipe Labellisée LIGUE Contre le Cancer, Institut Pasteur, CNRS UMR3738, 25 rue du Docteur Roux, 75015 Paris, France; 2 Sorbonne Université Collège Doctoral, F-75005 Paris, France; 3 Imaging and Modelling, Department of Cell Biology and Infections, Institut Pasteur, CNRS UMR 3691, 25 rue du docteur Roux, Paris, 75015 France; 4 Transcriptome and EpiGenome, BioMics, Center for Innovation and Technological Research, Institut Pasteur, 28 rue du docteur Roux, 75015 Paris, France; 5 Bioinformatics and Biostatistics Hub–C3BI, Institut Pasteur, CNRS USR 3756, 28 rue du docteur Roux, Paris, 75015 France

## Abstract

Transcription factor networks, together with histone modifications and signalling pathways, underlie the establishment and maintenance of gene regulatory architectures associated with the molecular identity of each cell type. However, how master transcription factors individually impact the epigenomic landscape and orchestrate the behaviour of regulatory networks under different environmental constraints is only partially understood. Here, we show that the transcription factor Nanog deploys multiple distinct mechanisms to enhance embryonic stem cell self-renewal. In the presence of LIF, which fosters self-renewal, Nanog rewires the pluripotency network by promoting chromatin accessibility and binding of other pluripotency factors to thousands of enhancers. In the absence of LIF, Nanog blocks differentiation by sustaining H3K27me3, a repressive histone mark, at developmental regulators. Among those, we show that the repression of *Otx2* plays a preponderant role. Our results underscore the versatility of master transcription factors, such as Nanog, to globally influence gene regulation during developmental processes.

## Abstract (web summary)

Transcription factor (TF) networks are essential for the molecular identity of each cell type. Here, the authors show that TF Nanog utilises multiple molecular strategies to enhance embryonic stem cell self-renewal, which include regulation of chromatin accessibility in the presence of LIF or maintenance of H3K27me3 at developmental regulators in its absence.

## Introduction

Gene regulatory networks driven by master transcription factors (TFs) play pivotal roles over a large spectrum of biological processes, from adaptive cell responses^1^ to cell fate specification during development^2^. The key properties of TF networks, shared among cell types, developmental contexts and organisms^3^, are exemplified by the pluripotency network, which plays a dominant role during early mammalian embryogenesis^4^. The robustness of this network allows to capture the ex vivo of transient biological identity of the pluripotent epiblast through the derivation of self-renewing Embryonic Stem (ES) cells^5^, which have enabled identification of key TFs (e.g., Oct4, Sox2, Nanog and Esrrb). The study of processes driving the balance between ES cell self-renewal and differentiation has provided us with a canonical picture of how TF networks operate, establishing self-sustaining regulatory loops and acting together through multiple promoters and enhancers^6–9^. For instance, Oct4, without which pluripotent cells cannot be maintained^10^, acts with the TF Sox2 to recognise and bind chimeric motifs^11^ found at a large number of regulatory elements driving ES cell-specific transcription. Oct4 and Sox2 also tend to bind with other TFs, including Nanog and Esrrb, at multiple enhancers across the genome, to combinatorially coregulate a large number of targets. This simultaneous and concerted action over hundreds of common targets ensures extensive redundancy, and, therefore, robust genome-wide responses. How these TFs synergise at or compete for common regulatory elements, and how by these means they individually contribute to the network’s activity, is however not well understood. Moreover, several TFs of the pluripotency network are directly connected to cell signalling, enabling ES cells to establish appropriate responses that are instructed extrinsically. A prominent example is provided by the LIF cytokine, which promotes self-renewal by activating several pluripotency TFs such as Esrrb^12,13^. Hence, a key function of the pluripotency network is to integrate signalling cues to appropriately respond to changes in the environment, conferring the responsiveness of ES cells and their capacity to readily differentiate. In this regard, it is noteworthy that *Nanog* was first identified as a factor capable of bypassing the requirements for LIF: in the presence of ectopic Nanog expression, ES cell self-renewal is strongly enhanced and completely independent of LIF^14^. In the current model, Nanog achieves LIF-independent self-renewal by activating LIF-responsive genes, in particular *Esrrb*. Hence, the Nanog-Esrrb axis and its intersection with LIF signalling represents a major mechanism by which intrinsic and extrinsic cues fine-tune self-renewal and avoid differentiation^15^. Yet, the precise mechanisms by which Nanog, and more generally the pluripotency network, controls differentiation genes are not fully understood. It is known, however, that differentiation genes adopt a particular chromatin state known as “bivalent”^16,17^: while their promoters are enriched for H3K4me3, a mark of gene activity, they are simultaneously embedded within larger domains of H3K27me3, a repressive mark. During differentiation, this state is resolved in either H3K27 or K4me3 in a lineage-specific manner^18^. In agreement, Polycomb Group proteins triggering H3K27me3 ensure appropriate cell fate changes^19–21^. This underscores the importance of H3K27me3 as cells dismantle the pluripotency network, inhibit self-renewal and exit from pluripotency. Whether bivalent chromatin marks are governed by pluripotency TFs remains to be thoroughly addressed.

In this study, we explore the function of Nanog in mouse ES cells using inducible approaches of gain-of-function and loss-of-function. We show that Nanog drives the recruitment of Oct4, Sox2 and Esrrb at thousands of regulatory regions, from where it mainly activates transcription. At these sites, Nanog also recruits Brg1 and promotes chromatin accessibility. On the contrary, to repress transcription Nanog does not recruit these TFs; rather, it frequently inhibits Oct4 or Sox2 binding. Nanog also binds at other enhancers where it acts redundantly with other TFs. However, in the absence of LIF the action of Nanog over these regulatory elements becomes dominant, particularly to promote transcription. This results in Nanog having an expanded action in the absence of LIF. Yet, its expanded repressive activity is not associated with ES cell enhancers. Rather, Nanog is required to maintain H3K27me3 at differentiation-associated genes. This is the case of the TF Otx2, whose downregulation by Nanog leads to LIF-independent self-renewal even when Esrrb is not expressed. Hence, Nanog deploys distinct molecular means to promote self-renewal and counteract differentiation: when the network is fully operative (in the presence of LIF), Nanog rewires its activity; when it is partially dismantled (in the absence of LIF), Nanog represses differentiation genes via H3K27me3. Overall, we reveal different modes and the varied logic employed by Nanog to orchestrate the three main features associated with self-renewal: the inter-dependencies between pluripotency TFs, LIF signalling, and bivalent chromatin domains.

## Results

### Inducible CRISPR-ON ES cells to activate *Nanog* transcription

**Fig. 1:**
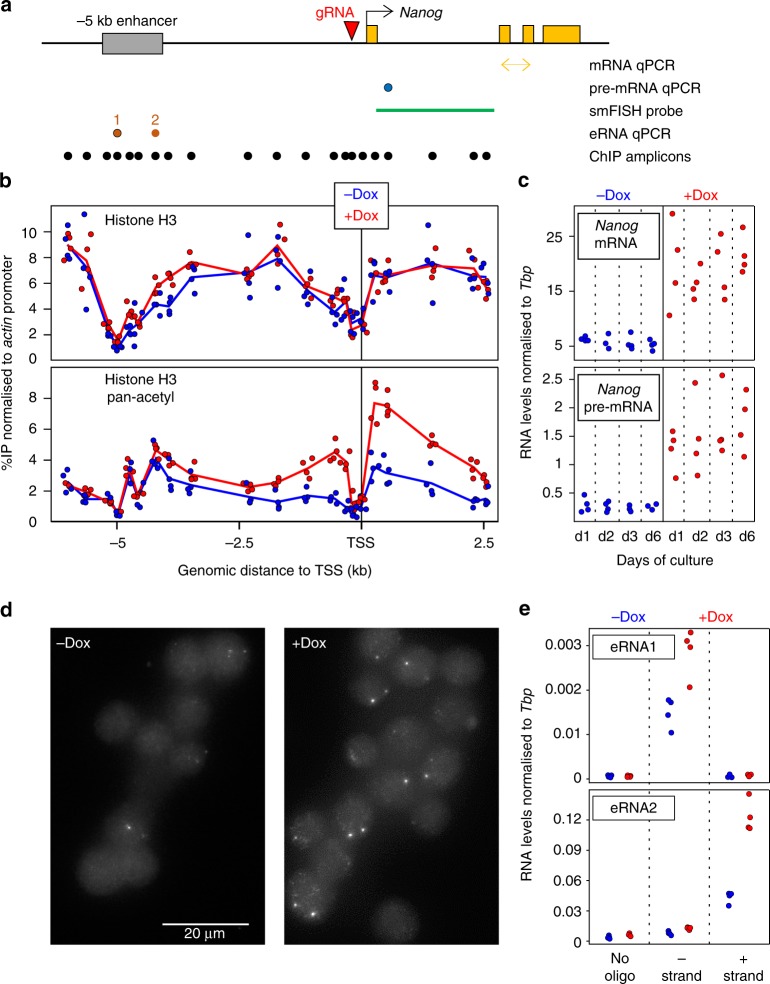
CRISPR-ON ES cells for Dox-inducible activation of endogenous Nanog. **a** Schematic representation of the *Nanog* locus (black arrow: promoter; yellow boxes: exons; grey box: Nanog enhancer; red arrowhead: gRNA). Below, the position of the amplicons and probe used for the assays indicated on the right is shown. **b** ChIP across the Nanog locus monitoring total histone H3 (top) and pan-acetyl H3 (bottom) in the absence (blue) and after 72 h of Dox treatment (red). Each dot represents normalised %IP measured in individual replicates and lines the averages. **c** Normalised levels of *Nanog* mRNA (top) and pre-mRNA (bottom) after the indicated number of days in the absence (blue) and the presence (red) of Dox. Each dot represents measurements in individual replicates as in **b**. **d** Representative smFISH image using intronic *Nanog* probes before and after 72 h of Dox induction. **e** Normalised levels of eRNA production from the −5 kb enhancer presented as in **c**. In all panels, *n* = 4; 2 with each independent SunTag clone

The SunTag system was developed as a versatile tool to either visualise specific molecules in live cells or to perform epigenome editing of endogenous loci when coupled to an enzymatically inert dCas9^22^. It involves the expression of diffusible antibodies (scFv) that interact with high affinity with 10 copies of the GCN4 epitope linked to an enzymatically inert Cas9 (dCas9). These scFv antibodies are fused to GFP and the potent activator VP64, such that upon expression of a gRNA targeting a given genomic region, several VP64 molecules are brought about with high efficiency and specificity. To provide increased flexibility to the system, and facilitate the generation of cell lines carrying an inducible CRISPR-ON system, we engineered a single vector expressing the two SunTag moieties under the control of a Tetracycline Responsive Element. Moreover, dCas9 is linked to BFP and HpH through P2A and IRES sequences, respectively (Supplementary Fig. 1A). Hence, upon induction of the system with Doxycycline (Dox), the cells are expected to become green, blue and Hygromycin-resistant, providing a high tractability. This vector was introduced in ES cells together with the rtTA activator: two clones (C1 and C2) showing a high percentage of green/blue cells upon Dox treatment and a strong induction of dCas9 and VP64 (Supplementary Fig. 1B, C), were selected. They both self-renew normally and differentiate in the absence of LIF; their karyotypes are also normal (Supplementary Fig. 1D). Next, we introduced to C1 and C2 a vector expressing a gRNA targeting the minimal *Nanog* promoter and validated binding of dCas9/VP64 with good specificity and inducibility (Supplementary Fig. 1E). This was accompanied by increased histone H3 acetylation around the promoter (Fig. 1a, b), as expected given the ability of VP64 to recruit histone acetyl-transferases^23^, in the context of presumably unaltered nucleosomal organisation as evaluated by total H3 analysis (Fig. 1b). Upon Dox induction, we observed efficient *Nanog* induction from 12 h of treatment onwards (Supplementary Fig. 1F, Fig. 1c), leading to an increase of Nanog protein levels (Supplementary Fig. 1C, G). We also found that the increase of *Nanog* expression was due to both stronger and more frequent transcriptional bursts (Fig. 1d and Supplementary Fig. 1H, I). Finally, we analysed the effects of Dox administration at the proximal −5 kb enhancer of *Nanog*: upon induction, we found both sense and anti-sense enhancer transcription to be increased (Fig. 1e). Whether this is due to the proximity of these two regulatory elements or to a functional influence of the promoter on the enhancer, remains to be determined. In conclusion, we have generated Dox-inducible SunTag ES cells to activate endogenous promoters and dissect the subsequent consequences.

### Definition of Nanog responsive genes

**Fig. 2:**
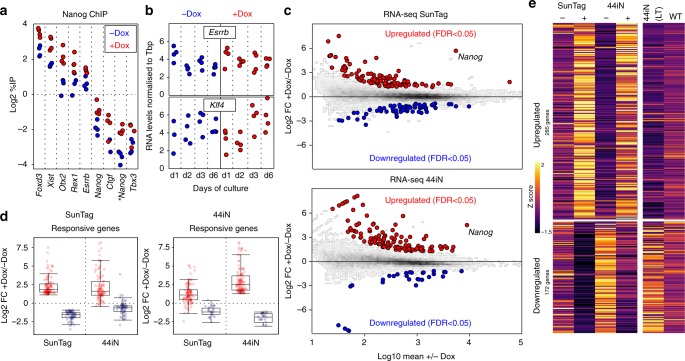
Identification of Nanog-responsive genes. **a** ChIP analysis of Nanog binding across a set of targets, as indicated on the *X*-axis. Each dot represents measurements in individual replicates (*n* = 4; 2 for each independent SunTag clone). **b** Normalised levels of *Esrrb* (top) and *Klf4* (bottom) mRNA after the indicated number of days in the absence/presence of Dox. Each dot represents measurements in individual replicates as in **a**. **c** MA Plots displaying log2 fold changes as indicated on the *Y*-axis as a function of average expression. RNA-seq was performed in both SunTag (72 h Dox induction) and 44iN cells (24 h Dox withdrawal). Red and blue represent differentially expressed genes (FDR < 0.05). **d** Boxplot of the log2 fold change for the genes identified in **c** as upregulated (red) or downregulated (blue) by Nanog, in either SunTag (left) or 44iN cells (right), measured in both inducible systems as indicated on the *X*-axis. For each boxplot, the central line represents the median, the limits the lower/upper quartiles, and the whiskers the most extreme data-point within 1.5 times the interquartile range in excess of the lower and upper quartile. **e** Heat map representing gene expression z-scores of all transcripts identified by combining SunTag (FDR < 0.05), 44iN (FDR < 0.05) and SunTag/44iN likelihood ratio test (FDR < 0.05) datasets

Upon Dox induction of *Nanog* in our SunTag cells we observed a 2-fold increase of Nanog binding to a panel of regulatory elements displaying a wide range of enrichment levels (Fig. 2a). This suggests that Dox induction may lead to functional consequences. However, the two main targets of Nanog that have been previously identified, *Esrrb* and *Klf4*^15^, showed minimal variation in expression levels, if any, over the course of 6 days of endogenous *Nanog* induction (Fig. 2b). Prompted by this unexpected observation, we performed RNA-seq to comprehensively study the global response to Dox treatment. We found a small number (163) of transcripts that were either upregulated or downregulated (Fig. 2c top); neither *Klf4* nor *Esrrb* were among the induced genes (Source Data, sheet 1; and Supplementary Fig. 2A, B). Nevertheless, the vast majority of genes that have been previously identified as responding to Nanog levels^15,24^, do exhibit the appropriate expression changes in our SunTag cells (Supplementary Fig. 2C). To further validate our list of Nanog-responsive genes, we performed a complementary analysis using previously established *Nanog*-null cells (44iN) expressing a Dox-inducible *Nanog* transgene^15^. The cells were grown in the continuous presence of Dox, which was then removed for 24 h leading to a nearly complete loss of *Nanog* expression (Fig. 2c bottom). The number of responsive genes observed with this strategy was also small (141; Fig. 2c and Source Data, sheet 1); they intersected with excellent statistical significance with the genes identified in the SunTag cells (Fisher *p* < 1e-53). Moreover, we found the expression of genes significantly regulated in only one system, to nevertheless display highly coherent expression changes in the other system (Fig. 2d). Hence, to improve statistical power and expand on Nanog targets we combined the SunTag and 44iN datasets to test for those genes with coherent Nanog response across both systems (Fig. 2e and Supplementary Fig. 2D). Combining with those genes already identified, this resulted in 457 genes, which generally display extremes of expression differences between Dox-treated SunTag (high *Nanog*) and untreated 44iN cells (low/absent *Nanog*; Fig. 2e and Supplementary Fig. 2D); they globally behave in a concordant way when long-term *Nanog*-null cells are compared to wild-type cells (Supplementary Fig. 2D), or when their expression is analysed in published data sets (Supplementary Fig. 2E). Genes activated by Nanog are enriched in regulators of stem cell maintenance (FDR < 2.99e−16), while repressed genes are enriched in differentiation processes such as nervous system development (FDR < 6.89e−10).

### Nanog rewires the pluripotency network to control its targets

**Fig. 3:**
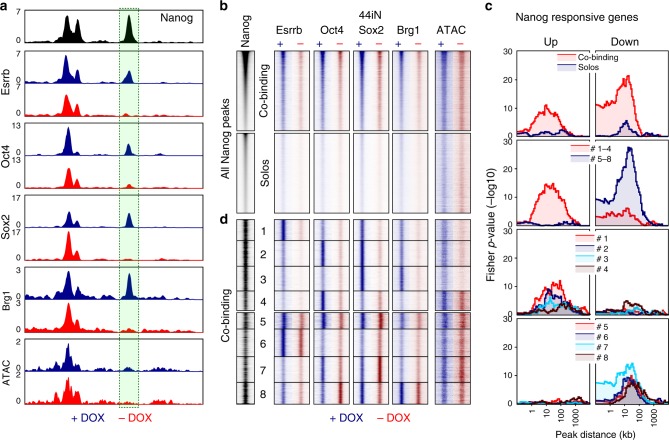
Nanog rewires the pluripotency network. **a** Representative enrichment profiles (reads per million) of the indicated TFs (ChIP-seq) and chromatin accessibility (ATAC-seq) across an 8 kb-long genomic region (mm9: chr10:33,952,000–33,960,000). In black, average signal from 6 publicly available Nanog datasets. In blue and red, average signal of 44iN cells cultured in the presence or absence of Dox, respectively. The peak highlighted by a green box shows reduced signal in the absence of Dox; note an upstream peak displays increased Sox2 binding. **b** Heat map of average enrichment levels in each indicated condition (+/−Dox) across 0.5 kb centred on the Nanog peak summit at all Nanog sites (27,782), ranked from high to low Nanog. The regions were split in two groups depending on the presence (co-binding) or absence (Solos) of other TFs. **c** Plots displaying the -log10 Fisher *p*-value (*Y*-axis) of the enrichment of genes upregulated (left) or downregulated (right), at a given distance (*X*-axis) from specific groups of Nanog binding sites identified in **b, d**, as indicated in the colour-coded insets. **d** Heat map corresponding to 8 clusters identified on the basis of TF co-binding and the effects of Dox withdrawal, presented as in **b** without ranking for Nanog binding levels

Having established a list of Nanog-responsive genes, we aimed at exploring the mechanisms by which Nanog influences their expression, focusing on a potential role of Nanog in modulating binding of other regulators such as pluripotency TFs (Oct4, Sox2 and Esrrb) and the chromatin remodeller Brg1 that is functionally associated with self-renewal^25,26^. To do this, we first established a list of 27,782 regulatory elements bound by Nanog using six data sets derived from four independent published studies (Source Data, sheet 2) and used 44iN cells to address how Nanog impacts TF binding and chromatin accessibility at these sites. We noticed that at some Nanog binding regions, Esrrb, Oct4, Sox2, and Brg1, display a strong reduction of binding and decreased chromatin accessibility, after 24 h of Dox withdrawal (Fig. 3a). This observation can be generalised to a large proportion of regions and is particularly prominent in the case of Esrrb (Fig. 3b). We then divided Nanog binding regions in two major groups (Fig. 3b and Supplementary Fig. 3), based on the presence of other TFs (regions of co-binding) or not (Nanog-solo regions). In Nanog-solo regions, which display lower levels of Nanog binding, the chromatin is less accessible irrespective of Nanog (Fig. 3b and Supplementary Fig. 3), indicating that additional factors may be recruited. Strikingly, when we computed the number of Nanog-responsive genes as a function of the distance to Nanog binding regions, we observed that the both activated and downregulated genes are particularly enriched in the vicinity of co-binding regions and not of Nanog-solos (Fig. 3c). Moreover, while activated genes tend to be located distally (within 10 to 100 kb), downregulated genes also show a significant enrichment over closer distances (<10 kb). To further explore the relationships between Nanog and other TFs, we used k-means clustering to identify 8 subgroups of Nanog binding sites (Fig. 3d and Supplementary Fig. 3). In the first 4 clusters, the depletion of Nanog leads to an acute loss of TF binding; collectively these regions are strongly associated with the activation of Nanog targets (Fig. 3c). In contrast, clusters 5–8 are significantly associated with genes repressed by Nanog and the effects of its depletion are more nuanced (Fig. 3c, d). More specifically, clusters 1 to 3 display a nearly total loss of TF binding in the absence of Nanog, along with a marked decrease in chromatin accessibility (Fig. 3d and Supplementary Fig. 3). These 3 clusters, in particular clusters 1 and 2, are associated with genes activated by Nanog (Fig. 3c). At cluster 4, however, chromatin accessibility shows minimal variations and, while very strong Oct4 binding is nearly completely lost upon Nanog depletion, Sox2 is not affected. Since Brg1 is particularly low across this cluster, Sox2 may recruit other chromatin remodellers to render the chromatin accessible at these regions, in a Nanog-independent manner. Accordingly, the correlation with Nanog-responsive genes of cluster 4 is weaker (Fig. 3c). Overall, at more than 6000 regions (clusters 1 to 3), Nanog plays a chief role in establishing functional and accessible regulatory regions capable of recruiting different combinations of TFs to activate its targets. Conversely, at clusters 5 to 8, the effects of the loss of Nanog are rather small both at the level of TF binding and of chromatin accessibility (Fig. 3d and Supplementary Fig. 3). This suggests that Nanog-mediated repression uses radically different mechanisms, which are not based on the increased recruitment of Esrrb, Oct4 and Sox2. Rather, clusters 7 and 8 display increased Oct4 and Sox2 binding in the absence of Nanog, respectively, suggesting that Nanog downregulates the genes functionally linked to these two clusters by blocking Oct4 or Sox2 recruitment (Fig. 3d and Supplementary Fig. 3). At other enhancers associated with genes repressed by Nanog, showing no alteration of Oct4 and Sox2 occupancy (clusters 5 and 6), Nanog may block the otherwise activatory function of other TFs. In conclusion, Nanog wires the pluripotency network by fostering TF recruitment and chromatin accessibility at distal regulatory elements to act as an activator, and uses different mechanisms, including the impairment of Oct4/Sox2 recruitment, both at promoter–proximal and distal regulatory elements of the genes it represses.

### Nanog-SunTag cells display LIF-independent self-renewal

**Fig. 4:**
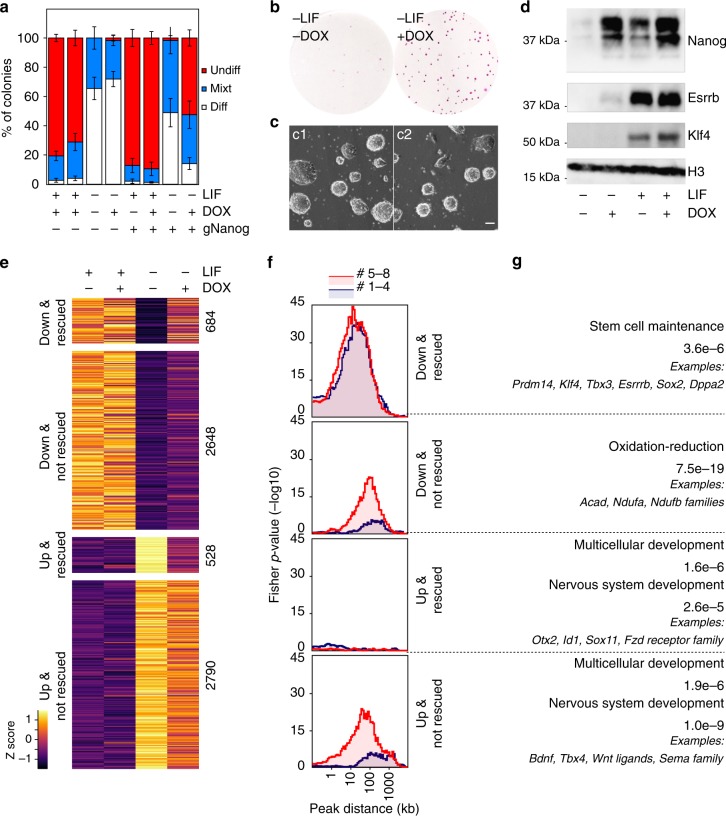
Endogenous induction of *Nanog* mediates LIF-independent self-renewal by non-canonical mechanisms. **a** Histogram representing the percentage of undifferentiated (red), mixt (blue) and differentiated (white) colonies (*Y*-axis) counted after 6 days of clonal growth in the indicated conditions (*X*-axis). Error bars represent std. dev. (*n* = 4). **b** Representative image of Alkaline Phosphatase stained colonies after 6 days of clonal growth in the presence/absence of Dox. **c** Representative image of the two SunTag clones after culturing them in 2i for 3 days following a 6-day clonal assay in the absence of LIF and the presence of Dox. The scale bar represents 50 µm. **d** Representative Western-Blot of Nanog, Esrrb and Klf4 after 3 days of culture in the indicated conditions. **e** Heat map representing gene expression z-scores across 4 groups of transcripts, as indicated in the left. UP/DOWN refers to their expression changes after 3 days of LIF deprivation (FDR < 0.05); rescued versus not rescued indicates whether Nanog significantly alleviates their misregulation (FDR < 0.05). **f** Plots displaying the -log10 Fisher *p*-value (*Y*-axis) of the enrichment of genes belonging to the groups identified in **e** at a given distance (*X*-axis) from Nanog binding clusters 1 to 4 (red) and 5 to 8 (blue), as established in Fig. 3. **g** Representative Gene Ontology terms enriched in each group of genes. The FDR is indicated together with selected examples

The strong influence of Nanog on the efficiency of self-renewal has been proposed to be largely mediated by Esrrb^15^. Therefore, we were not expecting our SunTag cells endogenously activating *Nanog* to exhibit increased self-renewal capacity, given that *Esrrb* and other genes involved in self-renewal, such as *Klf4*^12^, are not strongly induced (Fig. 2b). To test this, we initially plated our SunTag lines at clonal density together with the parental controls lacking the *Nanog* gRNA, and cultured them in the presence or absence of Dox for 6 days (Fig. 4a). In the presence of LIF, we could not observe any major change in the efficiency of self-renewal. In contrast, in the absence of LIF, when virtually all the colonies display complete or partial signs of differentiation in all controls, cells with enhanced endogenous *Nanog* expression generated a substantial proportion of undifferentiated colonies (Fig. 4a, b). To further validate that these cells are bona-fide ES cells, we harvested them at the end of the clonal assay and performed two complementary assays. First, we re-plated them in 2i medium lacking serum^27^, where only truly undifferentiated cells proliferate: both clones gave rise to typical spherical and undifferentiated colonies (Fig. 4c). Second, we re-plated them at clonal density in the absence of LIF and the presence/absence of Dox: only in the presence of Dox we did recover undifferentiated colonies; in the absence, all the cells differentiated (see below). This demonstrates that the exposure to Dox does not alter the differentiation capacity of our cell lines upon its withdrawal. We conclude, therefore, that Dox-induction of *Nanog* confers to our SunTag lines and the ability to self-renew in the absence of LIF, a definitive proof of the efficiency of our CRISPR-ON strategy to study Nanog function. Strikingly, LIF-independent self-renewal was attained in the absence of any apparent induction of *Klf4* and *Esrrb* mRNAs (Supplementary Fig. 4A) or proteins (Fig. 4d). Therefore, to explore both the magnitude of the differentiation blockade at the molecular level, and to identify potential Klf4/Esrrb-independent mechanisms underlying Nanog-mediated self-renewal, we performed transcriptomic analyses. In control cells that were not stimulated by Dox, a large number of genes responded to LIF withdrawal (>5000) and exhibited important quantitative differences (Supplementary Fig. 4B, C, D). In the presence of Dox, the magnitude of the expression changes of these LIF-responsive genes was globally diminished (Supplementary Fig. 4B), even though the vast majority of pluripotency genes remained strongly downregulated (Supplementary Fig. 4C, D). In fact, not all genes that respond to LIF withdrawal were rescued by *Nanog* induction to the same extent, with only around 20% being efficiently rescued (Fig. 4e, Supplementary Fig. 4E and Source Data, sheet 1). This argues against the idea that the presence of substantial numbers of undifferentiated cells may explain all the expression changes measured upon Dox induction in the absence of LIF.

### Increased regulatory potential of Nanog in the absence of LIF

In the absence of LIF, the effects of endogenous *Nanog* induction are largely maximised: if in the presence of LIF we identified 285 upregulated and 172 downregulated genes, in its absence these numbers raised to 856 and 589, respectively (Source Data, sheet 1). It appears, therefore, that LIF signalling attenuates the relative impact of Nanog on the ES cell transcriptome. To explore this further, we established the associations between the clusters of Nanog binding regions we have identified (Fig. 3), with four groups of genes: genes downregulated or upregulated upon LIF withdrawal and, among these two categories, those that Nanog can or cannot partially rescue by activating or repressing them, respectively (Fig. 4e). We observe that only one group, constituted of genes repressed by Nanog in the absence of LIF, is not enriched in any Nanog binding region that we have studied (Fig. 4f and Supplementary Fig. 4F). In contrast, the group of genes downregulated upon LIF withdrawal, and rescued by Nanog, is similarly enriched for Nanog binding regions where Nanog leads the recruitment of other TFs (clusters 1 to 4) than for those where it does not (clusters 5 to 8; Fig. 4f and Supplementary Fig. 4F). The activatory potential of Nanog through clusters 1 to 4 was already established from the previous analysis. However, the vast majority of genes upregulated by Nanog in the absence of LIF were not activated in LIF containing medium (>700 genes; Source Data, sheet 1). This suggests that in the presence of LIF, these genes were redundantly controlled by other LIF-dependent TFs, either through regions belonging to clusters 5 to 8 or through other regulatory elements where Nanog does not bind. Since clusters 5 to 8 were previously associated with genes repressed by Nanog in the presence of LIF, their enrichment in the vicinity of genes activated by Nanog in the absence of LIF implies that they are constituted by at least two functional categories: enhancers that are blocked by Nanog in the presence of LIF, leading to the downregulation of Nanog targets, and enhancers where Nanog also acts as an activator but redundantly to other LIF-dependent factors, most likely Esrrb. In agreement, this group is strongly enriched in genes of the pluripotency network (Fig. 4g), which are known to be controlled by several pluripotency TFs and from multiple distinct enhancers. The level of upregulation of these Nanog-activated genes is, however, relatively minor and the rescue of pluripotent TFs, including *Esrrb* and *Klf4*, marginal (Fig. 4d and Supplementary Fig. 4A, C). Finally, LIF-responsive genes that are not rescued by Nanog are associated with clusters 5 to 8 (Fig. 4f). This indicates that a large number of regulatory regions of the pluripotency network for which Nanog has a modest functional impact are present within these clusters. These regions activate or repress genes prior to differentiation in a Nanog-independent manner; upon LIF-withdrawal, their activity is likely invalidated with the ensuing consequences on gene expression even when *Nanog* is induced. These Nanog-independent, LIF-responsive genes, are closely associated with cluster 6 (Supplementary Fig. 4F), which is dominated by Esrrb (Fig. 3d and Supplementary Fig. 3), a prominent LIF target. This is particularly true for genes downregulated upon LIF withdrawal; satisfactorily, given the known role of Esrrb as a general regulator of metabolism and energy production^28^, these genes are enriched for related terms such as oxidation-reduction (Fig. 4g). Notably, the inability of Nanog to rescue these genes further supports the notion that our SunTag cells have acquired LIF-independent self-renewal in the absence of functional Esrrb. In conclusion, these analyses underscore the complexity of the pluripotency network: whilst Nanog activates genes both in the presence and absence of LIF through regulatory regions belonging to clusters 1 to 4, at other enhancers the activation of Nanog can only be unmasked in the absence of LIF, when other pluripotency TFs are downregulated and their functional redundancy with Nanog is abolished. Unexpectedly, however, the genes repressed by Nanog in the absence of LIF, which exhibit a robust rescue of higher magnitude than that observed for the genes that Nanog activates (Supplementary Fig. 4E), appear largely disconnected from the Nanog binding regions that we have studied here (Fig. 4f and Supplementary Fig. 4F). These genes are associated, among other categories, with signalling and molecular pathways linked to differentiation (Fig. 4g). Hence, the probably indirect repression mediated by Nanog over these genes may underlie LIF-independent self-renewal in Dox-treated SunTag cells, despite the lack of Esrrb and Klf4.

### Nanog sustains H3K27me3 upon LIF withdrawal

**Fig. 5:**
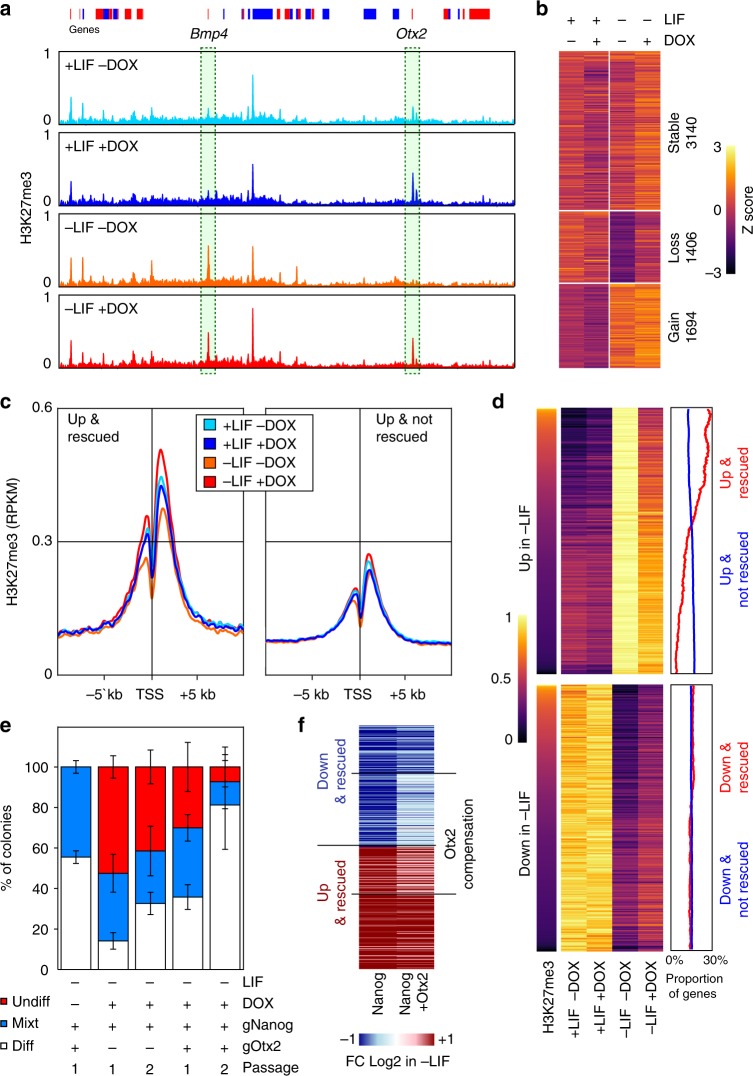
A Nanog/H3K27me3/Otx2 axis controls LIF-independent self-renewal. **a** H3K27me3 average levels (reads per million) across 5.15 Mb (mm9: chr14:45346123–50415313, red and blue boxes represent genes on the +and − strands) encompassing the *Bmp4* and *Otx2* genes that display the two stereotypical behaviours observed in SunTag cells cultured in the presence/absence of LIF/Dox for 3 days. **b** Heat map of H3K27me3 *z*-scores of 6,240 H3K27me3 domains identified in SunTag cells cultured as indicated. **c** Average H3K27me3 (reads per million) across promoter regions of genes upregulated in the absence of LIF and either rescued (left) or not (right) by Nanog. **d** Correlative analysis of ranked H3K27me3 (left) with gene expression changes (arbitrary units; middle) for transcripts upregulated (top) or downregulated (bottom) in SunTag cells cultured in the presence/absence of LIF/Dox for 3 days. The percentage of genes belonging to rescued (red line) versus non-rescued genes (blue line) in a 500 gene sliding window across the regions of the heat map is shown on the right. **e** Histogram representing the percentage of undifferentiated (red), mixt (blue) and differentiated (white) colonies (*Y*-axis) counted after 6 (Passage 1) or 12 (Passage 2) days of clonal growth in the indicated conditions (*X*-axis). Error bars represent std. dev. (*n* = 4). **f** Heat map of the average log2 fold change +Dox/−Dox in gene expression between SunTag cells activating either *Nanog* or *Nanog* and *Otx2*, grown in the absence of LIF and the presence/absence of Dox for 3 days. Genes for which the simultaneous activation of *Otx2* compensates the changes observed when only *Nanog* is induced (FDR < 0.05), are highlighted

Gene set enrichment analysis indicated that Nanog-rescued genes that are normally upregulated upon LIF withdrawal are enriched for targets of Polycomb Group proteins and for one of the marks they deposit to trigger facultative heterochromatin, H3K27me3 (FDR < 6e-43; Supplementary Fig. 5A). Hence, we profiled H3K27me3 in SunTag cells grown in the presence/absence of LIF and Dox. Overall, the patterns of H3K27me3 were found similar among all conditions, with notable exceptions (Fig. 5a). We identified three broad classes of H3K27me3 domains: those with high levels across all conditions and those that show either a loss or a gain of H3K27me3 upon LIF withdrawal (Fig. 5b and Source Data, sheet 3). Strikingly, the regions losing H3K27me3 in the absence of LIF maintained significant levels when endogenous *Nanog* expression was induced with Dox (Fig. 5b). In the presence of LIF, however, the induction of Nanog had minor consequences on H3K27me3, if any. This indicates that Nanog and LIF use parallel pathways to maintain H3K27me3 at a subset of H3K27me3 domains, and suggests that Nanog may confer LIF-independent self-renewal by sustaining H3K27me3 at these regions. Notably, the genes upregulated upon LIF withdrawal display differential enrichment among these three classes of H3K27me3 domains, depending on their Nanog-responsiveness: while nearly 70% of Nanog-rescued genes are marked by H3K27me3, which tends to decrease upon LIF-withdrawal except when Nanog is induced, only 30% of genes that are not rescued by Nanog show a similar pattern (Supplementary Fig. 5B). This confirms that Nanog-rescued genes are particularly enriched in LIF-dependent H3K27me3. More specifically, H3K27me3 concentrates around the promoters of these genes, and displays a reduction in levels upon LIF withdrawal, exclusively in the absence of *Nanog* induction (Fig. 5c). Hence, Nanog stimulates the maintenance of H3K27me3 at a large subset of the promoters it represses in the absence of LIF. Nevertheless, a third of the genes repressed by Nanog in the absence of LIF are not embedded within H3K27me3; conversely, a third of the genes that are upregulated upon LIF withdrawal regardless of Nanog expression are enriched in H3K27me3 (Supplementary Fig. 5B) and maintain higher levels around their promoters in the presence of Nanog (Fig. 5c). Thus, we determined whether quantitative differences regarding the effect of Nanog over these groups could be measured. Among the genes repressed by Nanog, the higher magnitude of rescue is observed for those genes that are embedded in H3K27me3 (Supplementary Fig. 5C). Similarly, even though their gene expression changes upon Dox induction were not statistically significant, within the group of genes not rescued by Nanog, those enriched in H3K27me3 show a clear tendency to be downregulated (Supplementary Fig. 5C). A clear, global pattern can be inferred from these analyses: the ability of Nanog to rescue genes that are upregulated upon LIF withdrawal is directly correlated with H3K27me3 levels. Accordingly, ordering the heatmap of genes upregulated upon LIF withdrawal (regardless of the ability of Nanog to rescue them) by their enrichment levels for H3K27me3 in the presence of LIF and the absence of Dox, naturally orders the genes from efficient to poor rescue (Fig. 5d). Hence, H3K27me3 levels before LIF withdrawal are highly predictive of the efficiency of Nanog to block gene upregulation during differentiation. Overall, these analyses indicate that in the absence of LIF, Nanog mediates its repressive function by other means than those described in its presence (Fig. 3): by maintaining high levels of H3K27me3. Since Nanog binding regions were not strongly associated with these genes (Fig. 4f), it seems likely that this effect is indirect and possibly mediated by the upregulation of the Polycomb protein Phf19^29^, which is upregulated upon *Nanog* induction (Supplementary Fig. 6).

### Nanog represses *Otx2* to confer LIF independency

Among the genes downregulated by Nanog in the absence of LIF are several developmental TFs such as *Sox11*, *Id1* and *Pou3f1*, among others (Source Data, sheet 1). Therefore, it may be possible that LIF-independent self-renewal is attained by the simultaneous inhibition of several developmental pathways. However, within the list of Nanog-repressed genes characterised by Nanog-dependent H3K27me3, we could also identify *Otx2* (Fig. 5a and Source Data, sheet 3), a key regulator of the earliest stages of ES cell differentiation^30,31^. Several lines of evidence point to *Otx2* downregulation being an important mediator of Nanog function. First, *Otx2* has been already identified as an important negative target of Nanog^15,32^; accordingly, we observe its downregulation at the mRNA and protein levels upon *Nanog* induction (Supplementary Fig. 5D, E). Moreover, further expression analyses indicate *Otx2* expression which is closely controlled by Nanog levels (Supplementary Fig. 5F). Second, the genes that are upregulated in the absence of LIF and that are rescued by Nanog, are enriched in genes activated by Otx2, while non-rescued genes are not (Supplementary Fig. 5A). Third, the ectopic expression of Otx2 drives ES cells into differentiation^31,32^, even in the presence of LIF, as we show here using our SunTag system targeted to the *Otx2* promoter (Supplementary Fig. 5E and G). Therefore, it may be possible that its Nanog-mediated downregulation contributes to LIF-independent self-renewal in the context of our endogenous *Nanog* activation, despite the lack of strong upregulation of *Klf4* and *Esrrb*. To test this, we exploited the flexibility of the SunTag system to simultaneously activate *Nanog* and *Otx2* and perform clonal assays. Upon the additional induction of *Otx2*, the proportion of undifferentiated colonies decreased in the absence of LIF, compared to cells activating *Nanog* only, in particular after two successive rounds of clonal growth (Fig. 5e). These results clearly place Otx2 as a key factor that needs to be repressed by Nanog in order to obtain efficient LIF-independent self-renewal. Next, we performed transcriptomic analyses upon *Nanog*/*Otx2* induction in the absence of LIF to identify the set of genes that were effectively compensated by the action of Otx2. We observed that around 40% of the genes repressed by Nanog, and 70% of the genes activated by Nanog, displayed similar levels to control cells grown in the absence of LIF and Dox, when both *Otx2* and *Nanog* were induced (Fig. 5f). Hence, at the molecular level, *Otx2* induction partially compensates the gene expression changes induced by Nanog overexpression in the absence of LIF, underscoring the antagonistic effect of Nanog and Otx2 over a large set of common genes^32^, including developmental TFs such as *Sox3*, *Sox11*, *Id1* and *Pou3f1*, among others (Source Data, sheet 1), which tend to be expressed in somatic cells and more particularly in neuroectodermal derivatives. In combination with the previous section, these results indicate strongly that Nanog controls H3K27me3 at key nodes in the differentiation network, such as *Otx2*, to indirectly repress a large set of genes involved with differentiation.

## Discussion

**Fig. 6:**
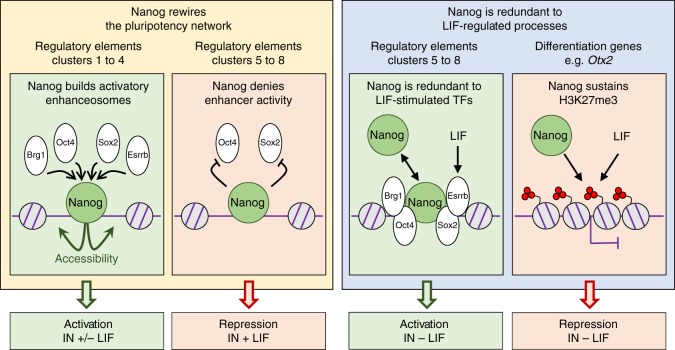
Nanog is a versatile TF impacting the pluripotency network and epigenome. The function of Nanog at stereotypical clusters of regulatory elements targeted by the pluripotency network, as well as at differentiation genes, is shown. Briefly, Nanog displays four major behaviours (left to right): 1/ recruitment of other factors (Oct4, Sox2 and Esrrb, together with Brg1) to promote chromatin accessibility and activate gene transcription; 2/ inhibiting enhancer activity, leading to gene repression either by blocking Oct4/Sox2 recruitment (shown) or by other mechanisms (not shown for simplicity; see text for details); 3/ complementing enhancer activity redundantly with other factors which are controlled by LIF (such as Esrrb)—in this case, its activatory role can only be appreciated in the absence of LIF; 4/ Nanog and LIF act in parallel to sustain H3K27me3 at differentiation genes such as *Otx2*. This latter role of Nanog is particularly important in the context of Nanog-mediated, LIF-independent self-renewal

Gene regulatory networks constituted of master TFs are characterised by the capacity of individual factors to act over the same sets of regulatory elements, which together define and specify the molecular and transcriptional identity of each cell type^33,34^. However, we still have a relatively poor understanding of how single TFs impact globally on the recruitment of other members of a given network to impact its activity. Recently, the role of Oct4 has been suggested to rely on its ability to recruit Brg1 to render the chromatin accessible for other TFs to bind^35^, matching a subset of the mechanisms we propose here for Nanog (Fig. 6). However, it is unclear how much the initiation of differentiation that follows Oct4 depletion^10^ influenced the interpretations regarding how Oct4 directly impinges upon the pluripotency network. In our case, we have focused on Nanog, a factor that can be depleted from ES cells while preserving pluripotency^36^. Hence, it is likely that the rewiring of the network that we observe shortly after depleting Nanog, is due to primary and direct effects. Strikingly, our analyses suggest that the simplified view positing that pluripotency TFs bind cooperatively at regulatory elements to collectively control transcription, may need to be partially revisited: at least from the perspective of Nanog, the combinations of binding, their dependencies on Nanog, and their association to responsive genes, are more complex than we had previously anticipated.

Although we observe, as expected, that Nanog-bound regions where other pluripotency TFs that are also recruited are more strongly associated with Nanog-responsive genes compared to regions where Nanog binds alone, we also find that only a small subset displays similarly high binding levels of all three pluripotency TFs that we tested (Esrrb, Oct4 and Sox2). Indeed, the binding of one or two factors, in addition to Nanog, tends to dominate the others. This is valid even for Oct4/Sox2, which are believed to bind together at Oct4/Sox2 chimeric motifs^11^. Therefore, different stoichiometries and/or residence times of individual factors seem to apply at distinct sets of regions. This produces a level of complexity that surpasses a simple model in which all factors bind together at key enhancers. Moreover, the effect of Nanog over these factors is also highly variable, with two clear groups of regions being easily identifiable: those in which Nanog plays a leading role; those where it does not (Fig. 6). Strikingly, these two groups of regions display sharp distinctions regarding their association to Nanog-responsive genes. Regions where Nanog preserves chromatin accessibility and drives the recruitment of other pluripotency TFs and Brg1, are strongly biased to genes activated by Nanog. Conversely, the regions where Nanog does not promote TF binding or accessibility are associated either with genes repressed by Nanog in the presence of LIF, or with genes where Nanog acts as a redundant activator with LIF-stimulated TFs.

To activate its targets, Nanog seems to use an expected mechanism essentially based on establishing a permissive chromatin architecture associated with the recruitment of other TFs and the formation of functional regulatory complexes. In contrast, while some precedents pointed in the direction of Oct4/Sox2-independent Nanog repression^37,38^, it is remarkable that Nanog-mediated repression is so strongly associated with regulatory elements where Nanog does not facilitate Esrrb, Oct4 and Sox2 binding and the chromatin remains equally accessible irrespectively of Nanog. This differential association between gene activation/repression and the role of Nanog as a nucleation factor of functional complexes, suggests that Nanog displays cooperative binding primarily to promote gene expression. At repressive sites, Nanog may either render the whole complex repressive or inhibit its otherwise transactivation potential. Additionally, at a large number of regions, the loss of Nanog leads to increased binding of either Oct4 or Sox2. It is therefore questionable that Nanog/Oct4 and Nanog/Sox2 bind at the same time on the same DNA molecules, at least over these regions. Thus, caution must be taken when extrapolating molecular functions from generic binding profiles: even though Nanog/Oct4 and Nanog/Sox2 appear to bind together, the binding of Nanog is in fact detrimental to that of the other two factors. Remarkably, these regions are closely associated with genes repressed by Nanog, indicating that to repress its targets Nanog interferes with the binding or the activity of other pluripotency TFs (Fig. 6). Whether the alternate behaviours of Nanog is to activate or repress transcription represents a general rule or a specific property of Nanog, which should be thoroughly investigated.

The ability of Nanog to block differentiation and promote LIF-independent self-renewal, something that not every pluripotent TF, including Oct4 and Sox2, is capable of doing, represents a defining property of Nanog^14^. However, this is not a unique characteristic of Nanog: a plethora of additional TFs, exemplified by Klf4 and Esrrb, have been progressively identified and demonstrated to provide LIF-independent self-renewal^12,15^. Hence, over the years, the importance of Nanog has been somehow equilibrated with that of other TFs, most notably Esrrb, which can replace Nanog in several contexts^15,39^. More strikingly, Esrrb has been proposed to be an obligatory mediator of the promotion of LIF-independent self-renewal by Nanog^15^. Therefore, our observation of LIF-independent self-renewal in the absence of strong Esrrb upregulation, and of many other pluripotency TFs, is particularly enthraling. This is not the first time, however, that the role of a TF within the pluripotency network needs to be nuanced. Nanog itself was initially thought to be essential for germ cell development^36^ and for the induction of pluripotency via somatic cell reprogramming^40^, conclusions that were subsequently attenuated by the demonstration that Nanog can ultimately be bypassed^39,41–43^. Moreover, major experimental differences between our and previous studies may underlie the different conclusions regarding the mandatory requirement for Esrrb. Indeed, while others ectopically expressed Nanog constitutively and at high levels in *Esrrb* knock-out cells^15^, we have used an inducible CRISPR-ON system to activate endogenous *Nanog* concomitantly with LIF withdrawal and the ensuing progressive downregulation of *Esrrb*. Therefore, the dynamic aspects of the two experimental setups are drastically different: it is possible that *Esrrb* is downregulated after Nanog has already impacted on other genes, which may independently block differentiation even when *Esrrb* is subsequently silenced. Besides this, however, our findings in the context of previous conclusions underscore that different factors and mechanisms can potentially lead to similar phenotypic outcomes^44^.

Identifying the genes that mediate LIF-independent self-renewal in the absence of Esrrb may be particularly challenging because several prominent developmental regulators, from TFs to signalling molecules, are enriched among the genes that normally respond to LIF withdrawal but that endogenous *Nanog* induction is able to block. However, the genes that are upregulated when LIF is removed, and that Nanog is able to keep in check, display a blatant property: they tend to be targets of Polycomb PRC2 complexes and are embedded within H3K27me3 domains^16,17,19^. At these genes, either LIF or Nanog are required to maintain H3K27me3 (Fig. 6). Therefore, this study identifies at least two modes of gene regulatory redundancy between Nanog and the LIF pathway: one directly based on LIF-stimulated TFs, such as Esrrb and Klf4, and another one based on the activity of Polycomb Group proteins. We anticipate that identifying the exact molecular mechanisms used by Nanog to modulate H3K27me3 will be of great interest, and propose here that they may be mediated, at least in part, by Phf19. Overall, our observations argue for the existence of an alternative pathway to promote LIF-independent self-renewal through a previously unanticipated role of Nanog in the maintenance of H3K27me3 at differentiation-associated genes, thereby inhibiting the capacity of the cells to readily differentiate. This type of compensatory, chromatin-based mechanism, enables individual TFs to have broad impact by targeting key chromatin regulators with a more generic and systemic function. This remains so even when a regulatory network is largely dismantled, as is the case of the pluripotency network in the absence of LIF. This mechanism that we have unveiled may have the potential to dramatically increase the robustness and temporal integration of complex gene regulatory systems.

Whether the promotion of LIF-independent self-renewal associated with the inappropriate maintenance of H3K27me3 at differentiation genes results from the sum of many partial effects or, conversely, is based on specific and potent effects mediated by one or a few regulators, needs now specific attention. Given that the genes repressed by Nanog in the absence of LIF are strongly enriched for targets of Otx2, a driver of differentiation^30,31^ that belongs to the category of bivalent genes in ES cells, we explored the possibility that *Otx2* downregulation may be the sole explanation for LIF-independent self-renewal in the absence of induced expression of other pluripotency TFs than Nanog itself. Using our SunTag cells to simultaneously activate both *Nanog* and *Otx2*, we could indeed observe that the efficiency of self-renewal was lower compared to the exclusive induction of *Nanog*. However, the effects became robust after two continuous phases of clonal growth, indicating that, for this level of upregulation of *Otx2*, the functional compensation takes time to be fully established. Nevertheless, our transcriptomic studies after three days of induction show that a large fraction of the genes normally misregulated upon *Nanog* induction have expression levels similar to control cells. In accord with the developmental role of Otx2^45^, this seems to be particularly true for neuroectodermal genes. It should now be investigated whether a relationship similar to Nanog-Otx2 exists between Nanog and other lineage specific determinants targeted by H3K27me3. Similarly, whether the Nanog-Otx2 functional interactions that we have studied also apply to additional TFs driving LIF-independent self-renewal requires additional work: the inducible SunTag system presented here will provide a flexible toolbox to address these and similar questions in mouse ES cells. Overall, this work underlines the ability of *Nanog* to convey its function through remarkably distinct molecular mechanisms in different environmental conditions (Fig. 6). Extrapolating our work to other TFs of the pluripotency network and more generally to other gene regulatory systems, would be of great interest.

## Methods

### Cell culture

*Regular cultures:* ES cells (E14Tg2a and derivatives) were cultured at 37 °C in 7% CO2 on 0.1% gelatine (SIGMA, G1890–100G) in DMEM + GlutaMax-I (Gibco 31966–021), 10% FCS (Sigma F7524), 100 μM 2-mercaptoethanol (Gibco 31350–010), 1× MEM non-essential amino acids (Gibco 1140–035) and 10 ng/ml recombinant LIF (MILTENYI BIOTEC, 130–099–895). Cells were passaged 1:10 every 2–3 days with 1× trypsin-EDTA 0.05% (Thermo 25300062). Only when mentioned cells were cultured in 2i medium containing 0.5× DMEM/F12 (Gibco 31331093), 0.5× Neurobasal (Gibco 21103049), 0.5% N2 supplement 100× (Gibco 17502048), 1% B27 supplement 50× (Gibco 17504044), 10 µg/mL Insulin (Sigma I1882–100MG), 2 mM L-Glutamine (Invitrogen 91139), 0.05% BSA (Sigma A3311–10G), 100 μM 2-mercaptoethanol (Gibco, 31350–010), 10 ng/ml recombinant LIF (MILTENYI BIOTEC, 130–099–895), 1 µM PD0325901 (Axon Medchem Bv Axon-1408), 3 µM CHIR99021 (Axon Medchem Bv Axon-1386). When specified cells were treated with 1 µg/mL of Doxycycline (Sigma D3072–1ML), 1 µg/mL Puromycin (Sigma P9620–10ML), 400 µg/mL Hygromycin B (Sigma H3274–50MG), 0.2 mg/mL G418 (Sigma G8168–10ML), 1 µM Tamoxifen (Sigma H7904–5MG).

*Nanog loss-of-function and gain-of-function:* For all SunTag induction experiments, 30,000 cells per cm^2^ were plated in presence or absence of Doxycycline/LIF. Medium was changed every day after one wash with the same medium. At day 3, cells were either lysed on the plate for RNA extraction or harvested for Western blot lysates, microscopy slides and chromatin preparation. For induction kinetics over 6 days, cells were passaged at day 3 at the same density. 44iN cells^15^ were kept in culture with Doxycycline for at least 3 passages, except when explicitly stated. Subsequently, 30,000 cells per cm^2^ were plated in presence or absence of Doxycycline for the indicated times. To culture 44iN cells in the absence of Nanog long-term, the cells were maintained under G418 selection, as previously described^15^. Finally, 44NERT^38^ cells were cultured under G418 selection and plated at 30,000 cells per cm^2^ to perform Tamoxifen induction kinetics for the indicated times.

*Clonal assays:* Clonal assays were performed by plating 600 cells/P6 well in +/− LIF and +/− Dox, in parallel. Medium was changed every day after one wash with the same medium. After 6 days, colonies were fixed (25% Citrate solution, Sigma 854: 67% Acetone, Sigma 270725; 8% Formaldehyde, Sigma F8775) for 1 min and stained for 20 min with Alkaline Phosphatase staining kit (Sigma, 86 R). Number of undifferentiated, mixt and differentiated colonies was then assessed on a stereo-microscope (NIKON-SMZ1500). For serial cloning assay, all cells from −LIF +Dox condition were harvested at day 6, counted and plated again at clonal density for 6 additional days, as indicated, and processed as above. Raw colony counts can be found as Source Data (sheet 4). Finally, to assess the pluripotent state of Nanog SunTag cells cultured in −LIF +Dox, all cells were harvested at day 6 and passaged 1:4 in FCS/LIF for 1 day to ensure correct cell adhesion. The next day medium was replaced by 2i/LIF.

### Generation of SunTag ES cells

*Cloning of the SunTag and gRNA expressing vectors:* The SunTag vectors were obtained from Addgene (#60903, #60904). The PiggyBac vectors containing the rtTA trans-activator (PB-CAG-rtTA) and a TRE-driven expression cassette (A-ND2), as well as the PBase vector were kindly provided by Dr. Pentao Liu^46^. To generate the PB-TRE-SunTag-dual-Hygro vector (see Sup. Fig1) expressing the two moieties of the SunTag system, we first modified #60903 as follows. The TRE promoter was PCR-amplified and inserted upstream of dCas9. Next, we excised the lentiviral part of the vector downstream of the WPRE element and ensured its integrity by ligating a short WPRE amplicon. Both 5′ and 3′ LTRs were PCR-amplified from PB-CAG-rtTA and sequentially inserted on both sides of the TRE-dCas9-GCN4-BFP cassette, and a bGH polyA signal was PCR amplified and inserted downstream of the BFP sequence. Finally, an IRES-Hph (Hygromycin resistance) cassette was amplified by PCR and inserted in frame with the dCas9-GCN4-BFP cassette upstream of the bGH polyA signal, generating the PB-TRE-dCas9-GCN4-P2A-BFP-IRES-Hph-pA vector. To modify #60904 we first PCR-amplified the scFv-GCN4-sfGFP-VP64-GB1-NLS cassette and inserted it in A-ND2 vector downstream of the TRE promoter. After a subsequent PCR amplification of the resulting cassette, it was finally inserted in the modified #60903 vector, generating our final Dox-inducible SunTag construct. To generate the gRNA expression vector, we used the #51133 plasmid (Addgene) to introduce the U6-gRNA-PGK-PuroR-pA cassette in the PiggyBac backbone of the PB-CAG-rtTA. The resulting plasmid is referred to as PB-gRNA-Puro. This vector was used to clone annealed oligos corresponding to the 20 bp of the sgRNA sequence preceded by specific overhangs (5′-CACC and 5′TTTG).

*Establishment of Parental SunTag ES cells:* Subconfluent E14Tg2a cells were transfected with 5 µL of Lipofectamine 2000 (ThermoFisher), 0.8 µg of PB-CAG-rtTA, 0.8 µg of dual SunTag PiggyBac vector and 0.4 µg of the PBase vector. Upon Doxycycline treatment (1 µg/mL) the cells were selected with Hygromycin B for 10 days. Single clones were manually picked and expanded in absence of Doxycycline and Hygromycin B. After expansion and stock freezing, induction of GFP/BFP expression was analysed for 6 clones on a LSR II Fortessa (Becton-Dickinson). Data were analysed using the FlowJo software suite (Tree Star). The 2 clones showing the best percentages of GFP/BFP positive cells under Doxycycline treatment and an absence of fluorescent signals in absence of Doxycycline were kept for further experiment (C1 and C2). The karyotype of C1 and C2 cells were established using colcemid arrest (4 h; 100 ng/ml^−1^; Gibco, 15212–012), hypotonic shock (NaCitrate 0.017 M, KCl 0.03 M) and cold acetic acid–methanol (1:3) fixation at 4 °C. Fixed cells were spread by dropping on pre-heated glass slides, mounted (Vectashield; VectorLab, H1200) and imaged using a Nikon Eclipse X microscope equipped with: ×63 oil immersion objective (N.A1.4); LUMENCOR excitation diodes; Hamamatsu ORCA-Flash 4.0LT camera; NIS Elements 4.3 software. Chromosomes number was then counted manually with NIS Elements 4.3 software for a minimum of 20 randomly chosen cells per clone.

*gRNA design and selection:* To design gRNAs targeting the SunTag system to the Nanog and Otx2 promoters, we first identified all the potential targets of 20 nucleotides preceding a ‘NGG’ protospacer adjacent motif (PAM) on both strands. Those having a GC content between 35% and 85%, and not containing a stretch of 4 or more repeated nucleotides were kept. A potential efficiency score was calculated for each candidate guide given the sequence and using a predictive model, as described^47^. To control off-target effects and ensure DNA targeting specificity, the remaining list of sgRNA candidates was mapped on the complete mm9 mouse genome using the EMBOSS Fuzznuc tool, allowing various ambiguities and complex search patterns. As CRISPR-CAS9 efficiency depends on sgRNA-target similarity pattern^48^, candidates having off-targets with only 0, 1 or 2 mismatches were excluded, while off-targets with 5 mismatches or more were ignored, as well as off-targets not followed by a PAM. Off-targets with 3 or 4 mismatches were then sorted by the lowest number of off-targets with 3 mismatches in the 5′ end, then by the highest proportion of off-targets with 2 mismatches or more in the seed. The efficiency of promoter induction with candidate gRNAs was then experimentally tested by transient transfection and the best gRNA was kept for further analyses.

*Generation of Nanog-, Otx2- and Nanog/Otx2-SunTag ES cells:* C1 and C2 were lipofected as described above with 1 µg of PB-gRNA-Puro and 0.5 µg of PBase-expression vector, selected with Puromycin for 4 days and plated clonally under selection. Twelve clones (six originating from C1 and C2) were selected for RT-qPCR induction of either Nanog or Otx2. One clone from C1 and another one from C2 showing good induction levels were finally used for all experiments. For Nanog-Otx2 dual SunTag cells, Nanog-SunTag C1 and C2 cells were lipofected as above with Otx2 gRNA. Ten clones were then picked for each C1 and C2, expanded, and analysed for Otx2 induction. One clone originating from each transfection showing good induction levels of both Nanog and Otx2 were used for all experiments.

*Generation of Nanog-SunTag ES cells with reduced Phf19 expression:* Nanog-SunTag ES cells derived from C1 parental clones were electroporated either with one of two shRNA expressing vectors targeting *Phf19*, or with scrambled shRNAs^29^, and selected with 0,2 mg/ml of G418 for one week. Only one clone displayed efficient knock-down of *Phf19* compared to cells expressing a scrambled shRNA. This clone expresses the following shRNA:

CCGGCCTAGCCAGTATATTCGACTTCTCGAGAAGTCGAATATACTGGCTAGGTTTTTG.

### Microscopy

*Bright field microscopy:* Cell culture dishes pictures were taken on a Nikon Eclipse Ti-S inverted microscope equipped with: CFI S Plan Fluor ELWD ×20 objective; 89 North PhotoFluor LM-75; Hamamatsu ORCA-Flash 4.0LT camera; NIS Elements 4.3 software.

*Single-molecule RNA Fluorescent* In Situ *Hybridisation (smFISH):* Cells were washed in 1× PBS, trypsinized, pelleted, washed again in 1× PBS and resuspended in 2 mL of DMEM/FCS medium. Cells were fixed with 1% Formaldehyde (Sigma F8775) with slow agitation. Fixation reaction was stopped by addition of 300 µL of 1 M glycine (SIGMA G7126–500G) for 5 min. Cells were then pelleted at 4 °C, washed in cold 1× PBS, and pelleted again. Cells were resuspended in cold 1%BSA 1× PBS at 1 million cells/mL and cytospun at 400 rpm (Low acceleration) for 5 min on SuperFrost slides (Thermo J1800AMNT). Slides were air dried and stored in 70° EtOH at 4 °C. Each spot was incubated at 37 °C for 3 h with hybridisation cocktail (10% Formamide, 2× SSC buffer, 1 µg/mL BSA, 1 µL of E.Coli RNAs at 1 µg/mL, 1 µL of Nanog probe at 20 pmol/µL). The slides were washed 3 times in 2× SSC 10% Formamide for 30 min at 37 °C and mounted in Vectashield medium with DAPI (Vector-abcys H-1200). Nanog pre-messenger probe was designed using Stellaris Probe Designer version 4.2 on Biosearch Technologies website with the maximum masking level (5) and was synthetised by the same company. Image stacks (0.5 μm gap) were acquired using a Nikon Eclipse X microscope equipped with: ×63 oil immersion objective (N.A1.4); LUMENCOR excitation diodes; Hamamatsu ORCA-Flash 4.0LT camera; NIS Elements 4.3 software. The number of active transcription sites per cell was automatically counted with FISH-quant program^49^. We used ImageJ software to measure the intensity of the signal at active transcription sites. A line of 16 pixels, with constant orientation being kept over all images, was centred on each analysed spot. The function Analyse > Measure was used to get pixel intensities along the line. The intensity of each pixel was then normalised to the mean of the first and last pixel of each individual analysed spot. Spots were randomly selected until at least 40 had been quantified for each of the Nanog SunTag clones in both −Dox and +Dox conditions.

### RNA, protein and chromatin analyses

*Western Blot:* One million cells were lysed in 100 µL of Laemmli Sample Buffer (Biorad, 1610737) and 10 µL loaded in wells of 10X Mini Protean TGX gels 10 % (Biorad 456–1034). Migration was performed at 20 mA for 1h30 and transfer to nitrocellulose membranes (GE Healthcare RPN303D) was performed at 300 mA for 1 h at 4 °C. Ponceau (Sigma P7170–1L) staining was used to check loading homogeneity and a picture was taken with a ChemiDoc MP Imaging System (Biorad 1705062). Membrane was blocked in PBS-0.1% Tween20 (PBST) 5%BSA for 1 h at room temperature. Membrane was incubated with primary antibodies at 4 °C overnight in blocking buffer, washed 3 times for 5 min in PBST at room temperature and incubated in blocking buffer with secondary antibodies for 1ht at RT. Membranes were washed three times in PBST and revealed with Clarity Max Western ECL Substrate (Biorad 1705062). Chemoluminescence was imaged on a ChemiDoc MP Imaging System (Biorad 1705062). Membrane stripping was done with mild stripping buffer (water 0.15% glycine, 0.1% SDS, 0.1% Tween 20, adjusted to pH 2.2) for three washes of 5 min. Membrane was blocked again after stripping and processed as above. The antibodies used can be found in Supplementary Data 1; uncropped versions of the blot can be found in the Source Data (sheet 5).

*RNA extraction and Reverse Transcription:* Cells were lysed with 1 mL TRIzol (ThermoFisher) and RNAs extracted according to manufacturer’s protocol. To eliminate any genomic DNA contamination, this was followed by an additional DNAse I treatment (Qiagen 79254) for 20 min at 37 °C followed by phenol:chloroform purification. RNAs were resuspended in Ultrapure DNAse/Rnase Free Distilled Water (Thermo 10977035). Reverse Transcription was performed with 1 µg of total RNAs with random hexamers or specific primers for strand specific RT-qPCR (Supplementary Data 1; Roche 04379012001) following manufacturer’s protocol on a TM 100 Thermal Cycler (Biorad).

*Quantitative real-time PCR:* Real-time PCR reactions were performed in duplicates in 384-well plates with a LightCycler 480 (Roche) using 4.5 µL of LightCycler 480 SYBR Green I Master (Roche, 04707516001), 5 µL of sample and 0.25 µL of each primer at 20 µM in a final reaction volume of 10 µL. Standard and melting curves were generated to verify the amplification efficiency (>85%) and the production of single DNA species. PCR primer sequences are listed in Supplementary Data 1. The 2dCt method was used both for ChIP and RT-PCR analysis. For the former, all values were corrected to the input; for the latter, Tbp was used to normalise the data.

*Chromatin preparation:* Nanog SunTag cells (3.10^7^) used for H3K27me3 analysis, were resuspended in 3 ml PBS and crosslinked for 10 min at room temperature with 1% formaldehyde (Sigma F8775). Crosslinking was stopped with 0.125 M glycine for 5 min at room temperature. 44iN cells (3.10^7^) used for TF binding profiling were crosslinked in 3 ml of freshly prepared PBS-DSG 2 mM at pH 7.0 (Sigma, 80424–5 mg) for 50 min at room temperature with occasional shaking. After pelleting and washing in PBS, cells were incubated for 10 min in 3 ml PBS 1% formaldehyde (Sigma F8775), quenched with 0.125 M glycine. After fixation, cells were pelleted, washed twice with ice-cold PBS and resuspended in 6 ml of swelling buffer (25 mM Hepes pH 7.95, 10 mM KCl, 10 mM EDTA) freshly supplemented with 1X protease inhibitor cocktail (PIC-Roche, 04 693 116 001) and 0.5% NP-40. After 30 min on ice with occasional shaking, the suspension was centrifuged and resuspended in 450 μl of TSE150 (0.1% SDS, 1% Triton, 2 mM EDTA, 20 mM Tris-HCl pH8, 150 mM NaCl) buffer, freshly supplemented with 1X PIC. Samples were split in 3 (150 µL) and sonicated in 1.5 mL tubes (Diagenode) using a Bioruptor Pico (Diagenode) for 7 cycles divided into 30 s ON - 30 s OFF sub-cycles at maximum power, in circulating ice-cold water. After centrifugation (10 min, full speed, 4 °C), the supernatant was stored at −80 °C. Five microlitres was used to quantify the chromatin concentration and check DNA size (typically 200–500 bp).

*Chromatin immunoprecipitation (ChIP):* The chromatin was pre-cleared for 90 min on a rotating wheel at 4 °C in 300 μl of TSE150 containing 50 μl of pG Sepharose beads (Sigma, P3296–5 ML) 50% slurry, previously blocked with BSA (500 μg ml−1; Roche, 5931665103) and yeast tRNA (1 μg ml−1; Invitrogen, AM7119). Immunoprecipitations were performed overnight rotating on-wheel at 4 °C in 500 μl of TSE150. 20 µL was set apart for input DNA extraction and precipitation. 50 µL of blocked pG beads 50% slurry was added for 4 h rotating on-wheel at 4 °C. Beads were pelleted and washed for 5 min rotating on-wheel at 4 °C with 1 ml of buffer in the following order: 3 × TSE150, 1× TSE500 (as TSE150 but 500 mM NaCl), 1× washing buffer (10 mM Tris-HCl pH8, 0.25 M LiCl, 0.5% NP-40, 0.5% Na-deoxycholate, 1 mM EDTA), and 2× TE (10 mM Tris-HCl pH8, 1 mM EDTA). Elution was performed in 100 μl of elution buffer (1% SDS, 10 mM EDTA, 50 mM Tris-HCl pH 8) for 15 min at 65 °C after vigorous vortexing. Eluates were collected after centrifugation and beads rinsed in 150 μl of TE-SDS 1%. After centrifugation, the supernatant was pooled with the corresponding first eluate. For both immunoprecipitated and input chromatin, the crosslinking was reversed overnight at 65 °C, followed by proteinase K treatment, phenol/chloroform extraction and ethanol precipitation. The antibodies and the amount of chromatin used for PCR or sequencing analyses is indicated in Supplementary Data 1.

*Chromatin accessibility (ATAC):* 50,000 viable cells were washed with cold 1× PBS, pelleted by centrifugation for 5 min at 500×*g* at 4 °C, resuspended in 50 μl of transposition reaction mix (25 μl of Tagmentation DNA buffer, 2.5 μl Tagment DNA enzyme (Illumina) and 22.5 μl nuclease-free H2O) and incubated for 30 min at 37 °C. DNA was purified with a MinElute PCR purification kit (Qiagen),

### Libraries preparation and sequencing

*RNA-seq:* We used 0.5 μg of total RNA to purify polyadenylated mRNAs and to build an RNA library, using TruSeq Stranded mRNA Sample Prep Kit (Illumina, #RS-122–9004DOC), as recommended by the manufacturer. Directional libraries were checked for concentration and quality on DNA chips with the Bioanalyser (Agilent). More precise and accurate quantification were performed with sensitive fluorescent-based quantitation assays (“Quant-It” assays kit and QuBit fluorometer, Invitrogen). Samples were normalised to 2 nM and multiplexed. After denaturation using 0.1 N NaOH (5′ at room temperature), the samples were diluted to 9 pM and loaded on the flowcell. Sequencing was performed on the HiSeq 2500 sequencer (Illumina) in 65 bases V4 single-end mode.

*ChIP-seq:* Immunoprecipitated DNA was end repair in a total volume of 50 µL (sample 37.5 µL, 10 mM dNTPs 2 µL, NEB T4 ligase buffer 5 µL, NEB T4 polymerase 2.5 µL, NEB Klenow polymerase 0.5 µL, NEB T4 PNK 2.5 µL) and incubated for 30 min at 20 °C. After DNA purification (see below), A-tailing was subsequently performed in a total volume of 25 µL (sample 20 µL, NEB Buffer 2 2.5 µL, 5 mM dATP 1 µL, NEB Klenow 3′-5′ exo minus 1.5 µL) at 37 °C for 30 min. Illumina TruSeq adapters were used for libraries indexing, ordered from IDT Company with 5′ phosphate modification. Illumina adapters’ compatibility was checked with the online tool checkmyindex (checkmyindex.pasteur.fr) for multiplexing. Truseq adapters were annealed with Illumina Universal adapter at 20 µM each in 1× NEB Buffer 2 on a TM 100 Thermal Cycler (Biorad). Adapters ligation was performed in a total volume of 25 µL (sample 19.25 µL, NEB 10× T4 ligase buffer 2.5 µL, 0.2 µM adapter 1.25 µL, NEB T4 ligase 2 µL) at 16 °C overnight. After DNA purification, DNA was amplified in a total volume of 50 µL (sample 19.5 µL, Pico green 1 µL—1:10 in water; Life Technologies P7589—, 25 µL of KAPA HiFi HOTSTART Ready mix—NC0295239—, PCR 1.0 10 µM 1 µL, PCR 2.0, 10 µM 1 µL) on a LightCycler 480 (Roche). PCR primers are listed in Supplementary Data 1. Any sample reaching an absolute fluorescence value of 6 was taken out from the plate at the end of the last extension. Any library requiring more than 16 cycles of amplification to reach this level was discarded and reprepared. DNA was finally purified and the concentration was measured with a Qubit 4 Fluorometer (Thermo Q33226). Libraries quality check and size estimation were then performed on an Agilent 2200 Tape Station with High Sensitivity D5000 ScreenTape (Agilent Technologies, 5067–5592) and High Sensitivity D1000 Reagents (Agilent Technologies, 5067–5585) using 1–2 ng of material. Libraries were subsequently adjusted to equimolar concentration of 2 nM according to fragments average size and concentration prior mixing them for subsequent sequencing on the HiSeq 2500 sequencer (Illumina) in 65 bases V4 single-end mode.

*ATAC-seq:* Libraries were prepared as described^50^ but replacing NEB Next High-Fidelity 2X PCR Master Mix, by KAPA HiFi HotStart (KapaBiosystems KM2602) for PCR amplification, after determining the number of cycles needed by qPCR. The concentration and quality of the libraries were assessed as described above. Libraries were sequenced on the HiSeq 2500 sequencer (Illumina) in 65 bases V4 paired-end mode.

*SPRI beads preparation and DNA purification:* SPRI beads for DNA purification were prepared as follows: 50% (w/v) PEG 8000 stock was prepared by progressively pouring nuclease-free water on 12.5 g of PEG 8000 (Promega V3011) until reaching a total volume of 25 mL. 1 mL of vigorously resuspended Sera-Mag Magnetic Speed beads (Ge Healthcare 65152105050250) were washed three times with washing buffer (10 mM Tris HCl pH 8, 1 mM EDTA, 0.05% Tween 20) by vortexing (15 s) and supernatant removal with a PureProteom Protein G Magnetic Bead System 11740343 (EMD Millipore). Then, incomplete storage buffer was prepared (25 mL NaCl 5 M, 6.25 mL Nuclease-free water, 0.5 mL Tris base 1 M, 0.5 mL Disodium EDTA 0.1 M) and 1 mL was used to resuspend the beads after last washing step by vortexing 15 s. Resuspended beads were added to the rest of the incomplete storage buffer and the mix was vortexed for 30 s. 17.5 mL of 50% (w/v) PEG 8000 stock was then slowly added to the mix. Finally, 250 µL of Tween 20 were added and the mix was slowly inverted until solution homogenised. Beads were further aliquoted in 2 mL Eppendorf tubes and kept at 4 °C. Optimal Beads:sample ratios for precise DNA size recovery were finally assessed using a 50 bp DNA step ladder (Sigma S7025–50UG) and an Agilent 2200 Tape Station using High Sensitivity D5000 ScreenTape (Agilent Technologies, 5067–5592) and High Sensitivity D1000 Reagents (Agilent Technologies, 5067–5585). DNA purification steps were performed in round bottom 96 well plates (Thermo 611U96) using an Agencourt SPRI Plate Super Magnet Plate (Beckman Coulter A32782) at room temperature. DNA sample and beads solution were mixed (0.8 to 2:1 ratio) in the P96 well and left for 5 min and washed twice with 200 µL 70% EtOH. DNase-free water was added to the beads for DNA elution, with successive rounds of pipetting. After 5 min, the samples were repositioned on the magnetic plate for 5 min and the supernatant was collected.

### Informatic analyses

*Data and availability:* A summary of data collected and sequenced for RNA-seq, ChIP-seq along with public data used to identify Nanog binding regions is available in Supplementary Data 2. All data collected is available from GEO (GSE118898). Briefly, for *Nanog* induction RNA-seq between 2–4 replicates were sequenced per condition; for 44iN ChIP-sequencing we collected two replicates per factor for plus and minus Dox, and correspondingly 2 replicates per +/− Dox for ATAC-seq. For H3K27me3 ChIP-seq we collected and sequenced 4 replicates per Lif/Dox condition, some of which were excluded due to quality issues (see Identification of H3K27me3 Domains). To identify Nanog binding regions we combined Nanog ChIP-seq from four independent studies: GSE56312^51^; GSE11724^52^; GSE44288^53^; GSE55404^54^. To compare to previously published Nanog targets, we used published microarray data^15,24^. Phf19 ChIP-seq data was obtained from GSE41589^48^.

*ChIP-seq Data Processing:* For all ChIP-seq samples (Nanog public datasets, 44iN+/−Dox, H3K27me3) reads were aligned with Bowtie2^55^ in the mm9 genome, with options “-k 10” for all samples and additionally “-I 0 -X 1000–no-discordant–no-mixed” for paired-end samples. Reads were additionally filtered for those with a single alignment (mapping quality: 255) and an edit distance less than 4 (mean edit distance for paired reads). For Nanog and 44iN datasets distinct reads aligning with identical coordinates were treated as duplicates and collapsed to one.

*RNA-seq Data Processing:* Stranded RNA-seq reads were aligned to the mm9 genome using STAR^56^ and quantified by RSEM^57^ using the RSEM-STAR pipeline, with additional options “–seed 1618–calc-pme–calc-ci–estimate-rspd–forward-prob 0.0”.

*ATAC-seq Data Processing:* Paired end 65 bp ATAC-seq reads were trimmed by aligning read pairs to discover regions of reverse complementarity surrounded by Nextera sequencing adapters. Similarly to ChIP-seq processing, reads were aligned to mm9 genome using Bowtie2^55^ with options “-k 10 -I 0 -X 1000–no-discordant–no-mixed”, and filtered for reads with a single alignment mean edit distance less than 4 between read pairs. Heatmaps and meta plots were generated by marking ATAC-seq cut sites, left-most and right-most coordinates of each read with shifted inwards by 4 bp, as recommended^50^, and base pair of the cut site and the two surrounding were marked. Total cut-site signal was normalised for sequencing depth and averaged over replicates.

*Identifying Nanog binding regions from Public datasets:* Six independent samples from four studies were combined, as specified above. Peaks were called against relevant inputs/controls for all samples using MACS2^58^ with “callpeak -q 0.2 -g mm”, with the exception of ref. ^54^ in which controls were unavailable, in this case MACS2 was run without controls and peaks are called against a local background model. Peaks intersecting with the mm9 blacklist (ENCODE Project Consortium 2012) were excluded along with those on chrM and chrY. This resulted in 69,088 and 25,047 peaks for ref. ^51^, 17,950 and 10,347 peaks for ref. ^52^, 31,062 peaks for ref. ^53^, and 27,888 peaks for ref. ^54^. Combined, this resulted in 85,697 candidate Nanog binding regions, which were further filtered to those occurring in at least two independent samples resulting in 39,164 peaks. Reflecting that these regions are representative of Nanog binding, we found the fraction of reads in these peaks to be high over all samples: 17.1%, 23.9%^51^; 16.6% and 16.6%^52^; 12.8%^53^; 25.8%^54^. Finally, to focus on those regions with clear Nanog binding we additionally filtered peaks to those with a minimum height (averaged over all samples) of 1 read per million resulting in 27,782 peaks.

*Clustering of 44iN ChIP-seq*: ChIP-seq signal for Esrrb, Oct4, Sox2 and Brg1 was quantified over 1 kb (+/− 500 bp) centred on Nanog peaks. Our objective was to identify the set of regions with strong co-binding between at least one of the factors and Nanog. Employing preliminary clustering on the sequencing depth normalised signals from the four factors, we found regions segregated into those displaying nanog co-binding and nanog solo behaviour. We found that a peak height threshold of ~30 reads normalised to the mean sequencing depth (~2.7 reads per million) for at least one factor in one condition captured the co-binding versus solo distinction. Applying this threshold resulted in 13,515 nanog solo regions, and 14,259 nanog co-binding regions which were subject to further comprehensive clustering. To identify patterns of co-binding that do not depend of differences between occupancy between factors or globally between sites (i.e., to group together Nanog sites which have the same binding pattern at potentially different occupancy levels), we normalise each factor to the same mean occupancy, and then normalise by the maximum peak height over all factors at each region. We apply k-means clustering on the combined normalised signal from+/− 250 bp surrounding the summit of each Nanog peak for each factor. Formally, if $h_{ijk}^ +$ is the normalised read depth in + Dox for factor *i* at region *j* at position *k*, and $h_{ijk}^ -$ correspondingly for −Dox, then the trace over the entire region of length is $t_{ij}^ + = \left( {h_{ij1}^ + ,...,h_{ijm}^ + } \right)$and similarly $t_{ij}^ -$ for −Dox. The combined trace is denoted $t_{ij} = \left( {t_{ij}^ + ,t_{ij}^ - } \right)$. We calculate a mean +Dox occupancy score for each factor: $\sigma _i = \mathop {\sum }\limits_{j,k} h_{ijk}^ +$, and then denote the normalised trace over *n* factors $\tau _j = \left( {t_{1j}/\sigma _1,...,t_{nj}/\sigma _n} \right)$, finally denoting its max-normalisation as $\overline {\tau _j} = \tau _j/\mathop {{\max }}\limits_k \{ \tau _{jk}\}$ .

We then apply a *k*-means clustering with a Euclidean distance metric on the $\overline {\tau _j}$. K-means clustering was applied using the Clustering package in Julia. We selected *k* to identify regions where the binding of factors is either dependent or independent of Nanog binding. We found *k* = 8 to provide a good balance between summarising broad Nanog dependence and identifying complex co-binding relationships: when evaluating the cluster assignments for *k* and (*k* + 1), in the range 2 ≤ *k* ≤ 20, we found that the Rand Index^59^, defined as the number of pairs of regions assigned to the equivalent cluster over the total numbers of pairs exceeded 0.9 for *k* ≥ 7. Importantly, only at *k* = 8 the Oct4 only dependent cluster which retains accessibility (Fig. 3) was resolved, and for *k* = 9 and *k* = 10 the overall cluster identity in terms of dominant factors and dependent versus independent assignments no longer changed.

*Identification of H3K27me3 Domains:* Candidate H3K27me3 domains were identified by MACS2^58^, run in broad peak mode with options “–broad -q 0.2-g mm” against relevant inputs. A high level on concordance in peaks between the samples was observed. We merged all peak regions within 3 kb, taking those present in at least 8 samples (i.e., all four replicates of at least two conditions), to focus on broad domains. This resulted in 6240 H3K27me3 domains. We noticed that certain replicates were outlying, and excluded those samples that had the maximum reads per peak over all replicates of a condition in at least 60% of the peaks. This excluded a sample from each of SunTag + LIF-Dox, SunTag, + LIF + Dox and SunTag −LIF+Dox. We considered a gene to be embedded within a H3K27me3 domain, if its loci intersected with the domain by at least 1 bp or if a domain lay within 4 kb of the TSS and that domain did not intersect with another gene.

*Clustering of H3K27me3:* To identity a broad set of H3K27 domains with a dynamic response to removal of LIF and Nanog induction, we clustered the total number of reads per peak max normalised over all SunTag conditions by k-means clustering with *k* = 3.

*RNA-seq Differential Expression Analysis in the presence of LIF:* RSEM estimated read counts per sample were rounded for use with DESeq2^60^. Genes with at least 10 normalised counts in all replicates of at least one condition were considered for differential expression analysis. For all differential expression tests DESeq2 was run without independent filtering and without any fold change shrinkage, genes with FDR < 0.05 are considered differentially expressed. For +LIF samples, Nanog responsive genes in SunTag were identified by a Wald Test with the formula ~Dox on SunTag samples, and correspondingly the same was applied to 44iN samples. Since the overlap between the two systems is good (3 × 3 contingency table accounting for up, down, and non-significantly regulated *χ*² = 2126.97, df = 4, *p* ≈ 0.0 and a large statistical effect Cramér’s V = 0.267), and non-significant genes in either SunTag or 44iN had the correct fold change when the gene was significantly misregulated in the other setup (Supplementary Fig. 2), we tested to find those genes consistently mis-regulated by induction of *Nanog* in SunTag or 44iN by a Likelihood ratio test. More specifically, we tested the alternative hypothesis ~Cell + Dox + Cell:Dox over the null model ~Cell, where Cell is a factor indicating SunTag or 44iN and Dox indicating Dox treatment. The likelihood ratio test identified 419 Nanog responsive genes (FDR < 0.05), including 152/164 genes identified by SunTag alone, and 115/141 genes identified by 44iN alone. To select a list of Nanog responsive genes in +Lif we required that a gene with differentially expression with FDR < 0.05 in any of SunTag alone, 44iN alone or the likelihood ratio test, resulting in 457 genes.

*RNA-seq Differential Expression Analysis in the absence of LIF:* For SunTag Nanog RNA-seq in+/− LIF and +/− Dox we opted for Wald tests on contrasts on the formula ~LIF + Dox + LIF:Dox. This allowed us to identify genes that responded to LIF or Dox in independent manner along with genes whose response to Dox was dependent on LIF. We tested three variables: LIF, Dox and the sum of Dox and LIF:Dox interaction term, resulting in a fold change due to the loss of LIF, a fold change due to the addition of Dox in +LIF and a fold change due to the addition of Dox in −LIF. Classifying genes as either activated, repressed or not significant for each variable results in the assignment of genes to one of 24 different patterns of response. We tested 15,301 genes and found 7999 genes (52.2%) had no significant change in expression in either +/− LIF or +/− Dox; 2790 (18.2%) and 2648 (17.3%) genes were activated and repressed upon loss of LIF with no Dox response; 684 (4.7%) genes were repressed on loss of LIF and activated by Nanog in -LIF; 528 (3.4%). The remaining ~5% of genes either had a Nanog response in +LIF only, or a Nanog response in -LIF in which the gene did not respond to the removal of LIF. To assess the potential Otx2-driven compensation of Nanog effects, we applied an identical analysis to Nanog alone, noting those cases in which a gene previously assigned as a Nanog target in -LIF, is now either not significant or is significant in the opposing direction. To detect those genes where Otx2 only partially compensates for Nanog rescue (i.e., expression is not returned to −LIF −Dox levels), we combined Nanog and Nanog/Otx2 SunTag samples and tested ~LIF + DoxGuide + LIF:DoxGuide, where DoxGuide is a factor representing +/− Dox and either Nanog alone or Nanog/Otx2 guides. We tested for the difference between the two guides in +Dox -LIF.

*Gene Peak Proximity Enrichments:* To determine whether a set of Nanog responsive genes were enriched in proximity to a set of Nanog peaks. We calculated the distance between the TSS of each gene that had been tested for differential expression and the set of Nanog peaks. We then performed Fisher exact tests between the genes in the responsive set to all expressed genes within *x*bp of a peak, for *x* in the range [1, 1e + 8] bp.

### Reporting summary

Further information on experimental design is available in the Nature Research Reporting Summary linked to this article.

## Supplementary information

Supplementary Information

Description of Additional Supplementary Files

Supplementary Data 1

Supplementary Data 2

Reporting Summary

Source Data

## Data Availability

All genome-wide datasets are available through the NCBI Gene Expression Omnibus (GEO; https://www.ncbi.nlm.nih.gov/geo), under the accession number GSE118898. The data underlying our ChIP-seq, ATAC and RNA-seq analyses, the raw data underlying graphs, and uncropped versions of blots are provided as Source Data. All other relevant data supporting the key findings of this study are available within the article and its Supplementary Information files or from the corresponding author upon reasonable request. A reporting summary for this article is available as a Supplementary Information file.
