# Supplementary material for: The molecular logic of Nanog-induced self-renewal in mouse embryonic stem cells
Source: Nat Commun. 2019 Mar 7;10:1109. doi: 10.1038/s41467-019-09041-z (PMC6406003; doi:10.1038/s41467-019-09041-z)
Supplement: Supplementary file 1 — Supplementary Information [file 41467_2019_9041_MOESM1_ESM.pdf]

The molecular logic of Nanog-induced self-renewal in mouse embryonic stem cells

Heurtier & Owens et al.

Supplementary Information

Contents:

Supplementary Figure 1: Characterisation of Dox-inducible SunTag ES cells.

Supplementary Figure 2: Validation of Nanog-responsive genes identified in this study.

Supplementary Figure 3: Heterogeneity of TF binding and chromatin accessibility throughout Nanog binding regions.

Supplementary Figure 4: Global and gene-specific responses of the transcriptome to Nanog induction in the absence of LIF.

Supplementary Figure 5: H3K27me3 and Otx2 are involved in LIF-independent self-renewal as established by Nanog.

Supplementary Figure 6: Phf19 as a candidate Polycomb protein linking Nanog function to H3K27me3.

Available on-line:

Source Data File: RNA-, ChIP- and ATAC-seq data used in this manuscript, together with raw counts of clonal assays and uncropped versions of western blots.

Supplementary Data File 1: Primer and gRNA sequences as well as antibodies information.

Supplementary Data File 2: Global statistics of our sequencing data.

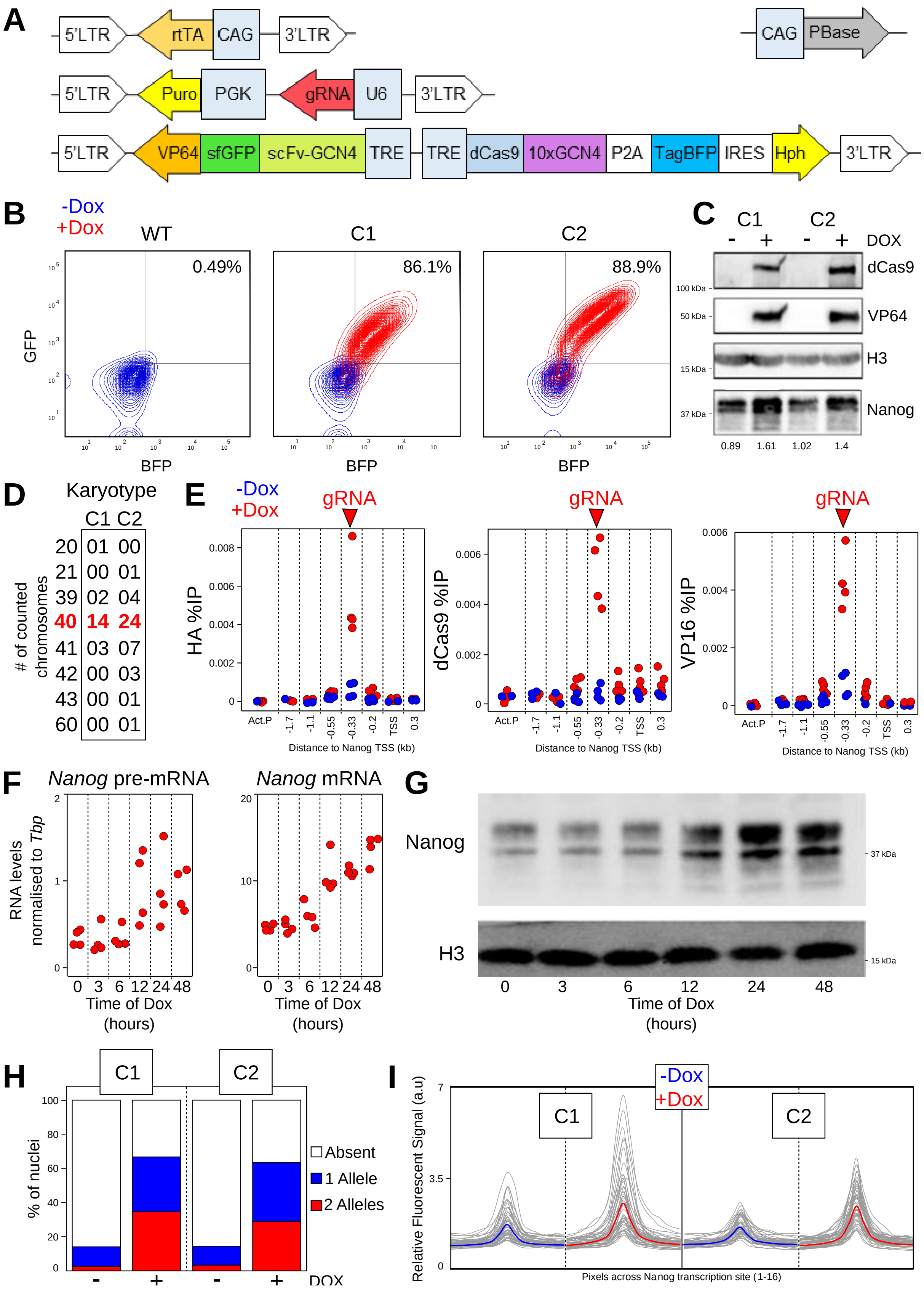

	C1	C2
20	01	00
21	00	01
39	02	04
**40**	**14**	**24**
41	03	07
42	00	03
43	00	01
60	00	01

Supplementary Figure 1: Characterisation of Dox-inducible SunTag ES cells. (A) Schematic representation of the Piggybac vectors used to generate ES cells expressing inducible SunTag transactivators. LTR: long terminal repeat; rtTA: reverse tetracycline-controlled

transactivator; PBase: Piggybac transposase (non-integrative vector); CAG: constitutive RNAPII promoter; Puro: Puromycin resistance cassette; PgK: constitutive RNAPII promoter; U6: RNAPIII promoter for gRNA transcription; VP64: tetrameric fusion of Herpes simplex virus transactivation domain; sfGFP: super-folder green fluorescent protein; scFv-GCN4: Single-chain variable fragment antibody directed against the GCN4 yeast epitope; TRE: tetracycline responsive element; dCas9: enzymatically dead Cas9; 10xGCN4: 10 copies in tandem of the yeast GCN4 epitope; P2A: self-cleaving peptide; TagBFP: monomeric blue fluorescent protein; IRES: internal ribosome entry site; Hph: Hygromycin resistance cassette. **(B)** GFP versus BFP FACS profiles of wild-type cells (WT) and of two SunTag clones (C1 and C2) in the absence (blue) and the presence of Dox (red). The percentage indicates the proportion of double-positive cells. **(C)** Western-Blot analysis of the indicated proteins (right) in the indicated conditions (top). The numbers underneath indicate relative Nanog levels. **(D)** Karyotypes of SunTag ES cells. **(E)** ChIP analysis of the indicated proteins (the HA epitope is present in the two moieties of the SunTag system) at different positions along the *Nanog* locus (X-axis) with (red) and without Dox (blue). The amplicon overlapping the gRNA targeted sequence is indicated (top). Each data point represents an independent replicate (n=4; in both C1 and C2). **(F)** RT-qPCR analysis of *Nanog* pre- and mRNA upon Dox induction. Each data point represents an independent replicate (n=4; in both C1 and C2). **(G)** Western-Blot analysis of Nanog protein upon Dox induction. **(H)** Proportion of SunTag cells displaying one or two *Nanog* active transcription sites as established by smFISH in the presence/absence of Dox for the two SunTag clones (n>500 nuclei in each clone/condition). (I) Relative quantification of the fluorescent signal measured along a line crossing *Nanog* transcription sites (n=40 sites in each clone/condition).

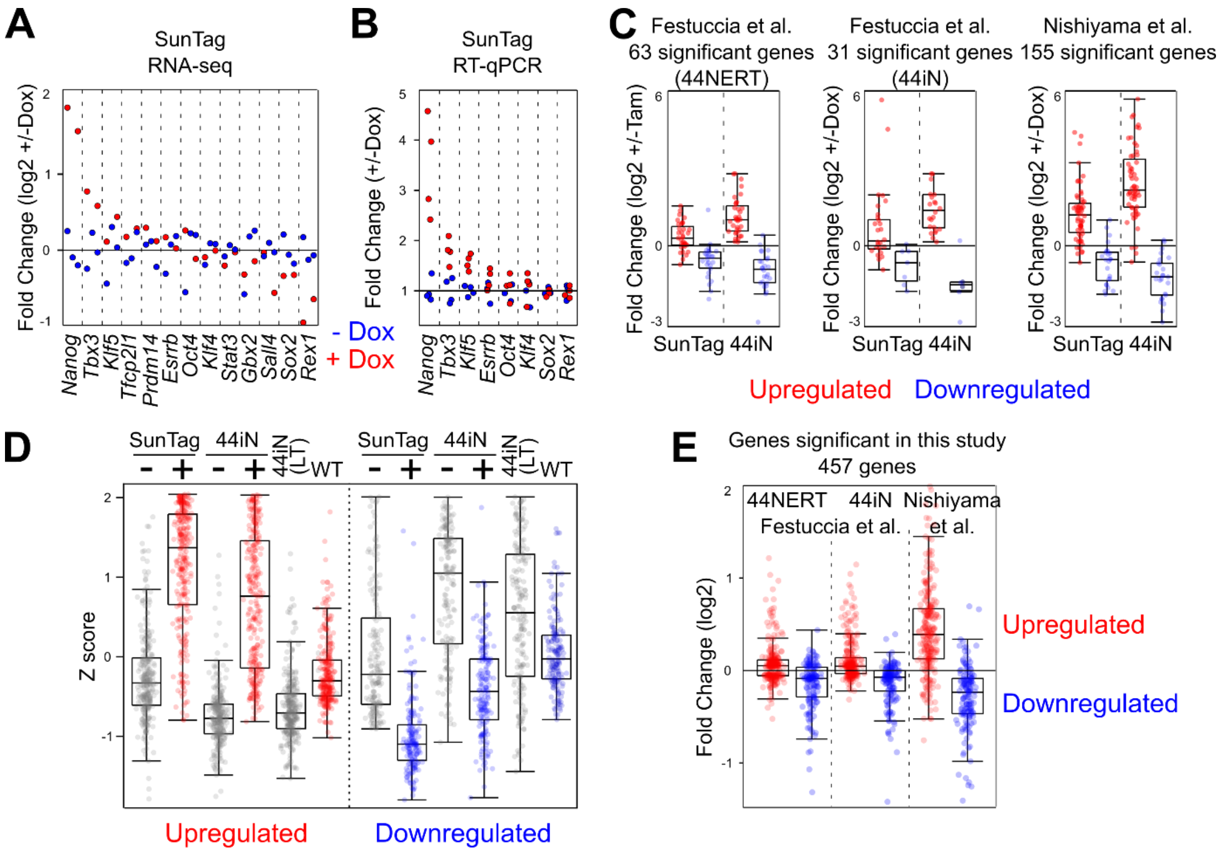

Supplementary Figure 2: Validation of Nanog-responsive genes identified in this study. (A) Fold change (log2) of a set of pluripotency genes upon Dox treatment of SunTag cells. For each transcript, each value in the absence (blue) and in the presence (red) of Dox was normalised to the average of independent replicates. **(B)** RT-qPCR validation of a subset of the transcripts analysed in (A). **(C)** Confrontation of published results, as indicated, with our Nanog-responsive genes identified in SunTag and 44iN cells. **(D)** Boxplot representation (z score, as in Figure 2E) of expression levels of the genes identified in this study when considering both SunTag and 44iN together. 44iN(LT) indicate long-term culture in the absence of Dox (i.e. *Nanog* knock-out). For each boxplot, the central line represents the median, the limits the lower/upper quartiles, and the whiskers the most extreme data-point within 1.5 times the interquartile range in excess of the lower and upper quartile. **(E)** Quantification in published datasets, as indicated, of our extended list of Nanog-responsive genes.

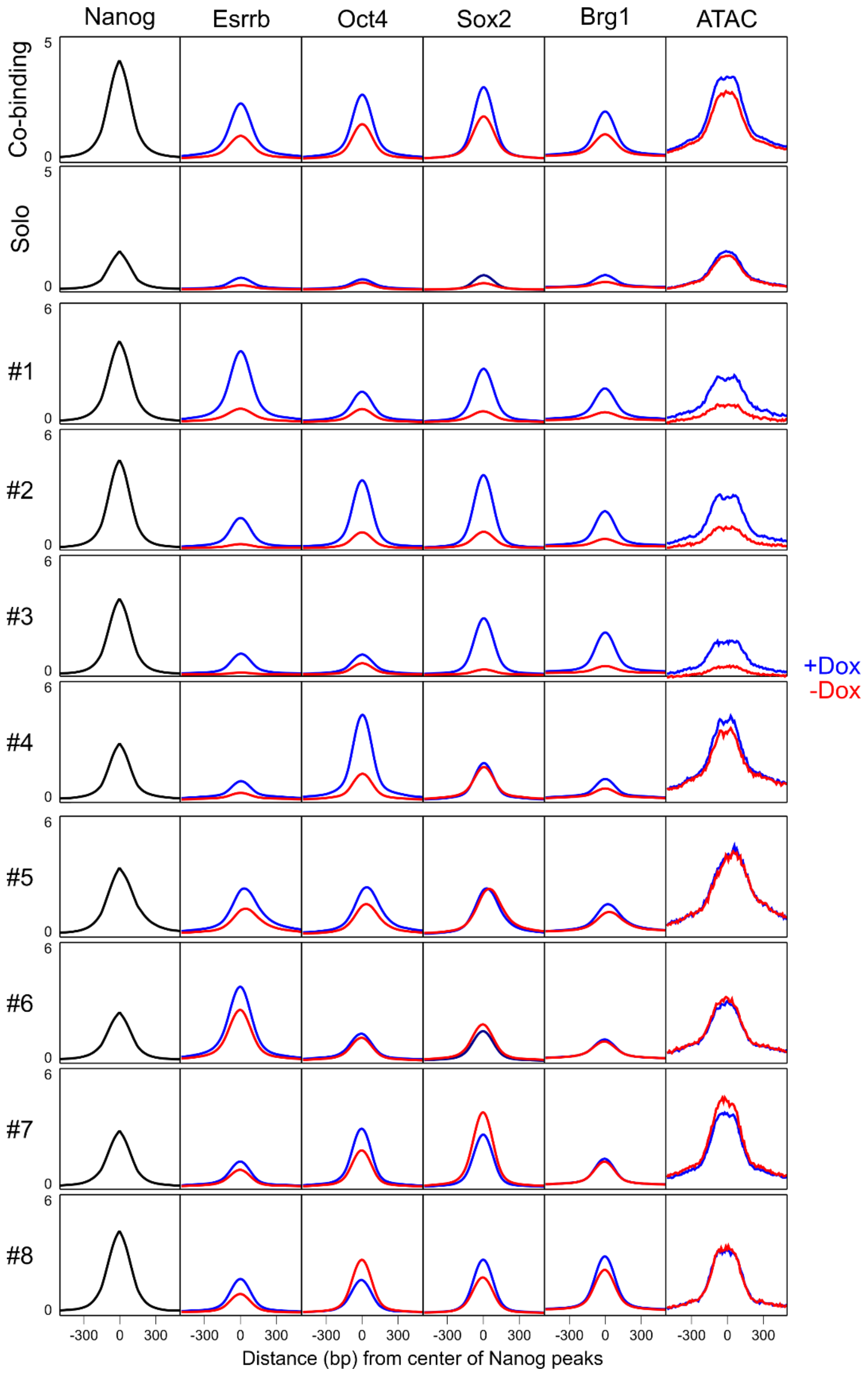

Supplementary Figure 3: Heterogeneity of TF binding and chromatin accessibility throughout Nanog binding regions. The average binding profile of each factor (A.U. correcting for TF occupancy as described in Methods), as labelled on the top, is shown across Nanog peaks (summit at 0bp) for each Nanog subgroup identified in Figure 3.

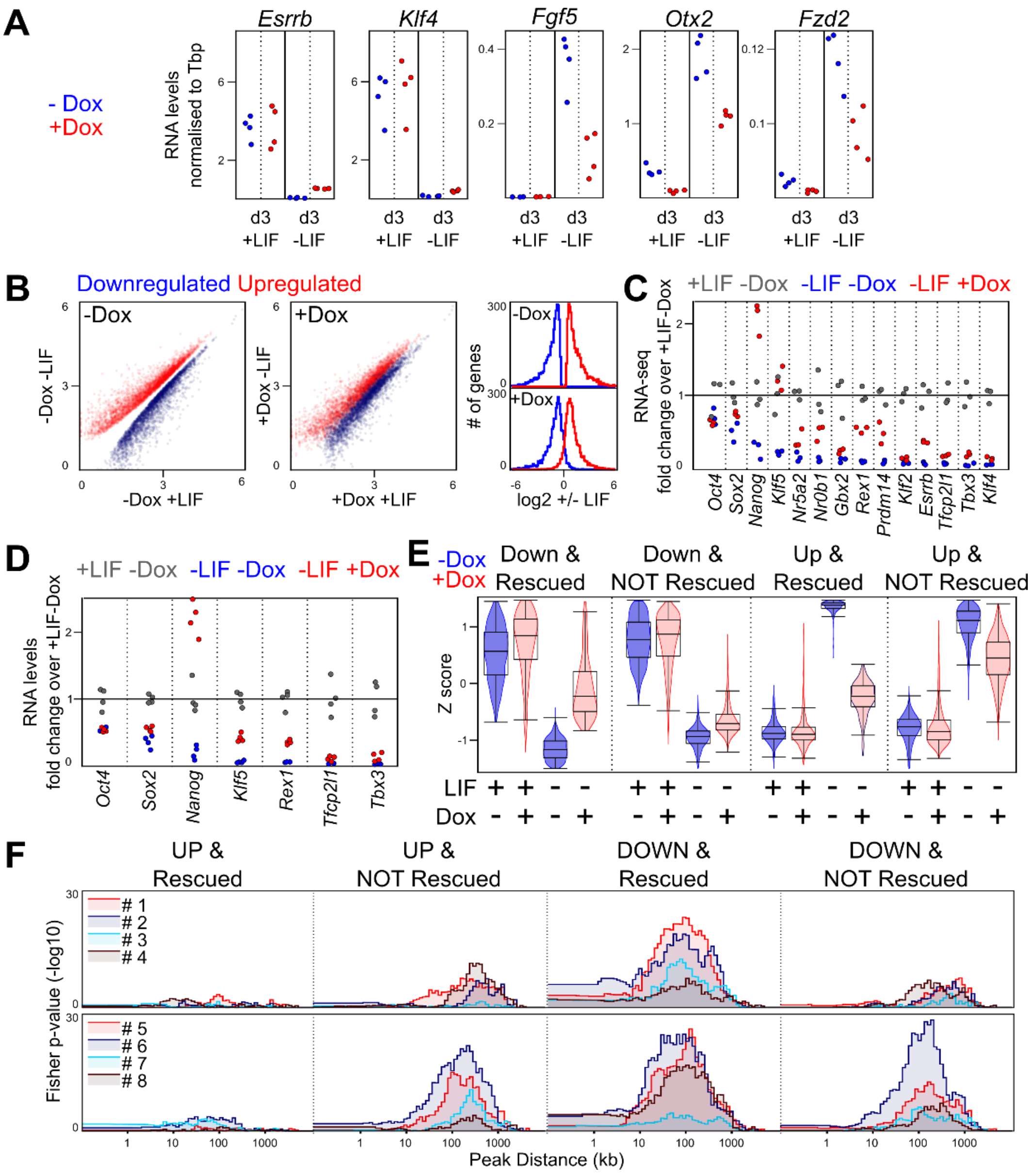

Supplementary Figure 4: Global and gene-specific responses of the transcriptome to Nanog induction in the absence of LIF. (A) RT-qPCR of the indicated mRNAs across the conditions shown on the X-axis, in the absence (blue) and the presence (red) of Dox. Each data point represents individual measurements in both C1 and C2 clones (2 for each). **(B)** Left: scatter plot of normalised mRNA levels (DESeq2 normalised) of differentially expressed genes in the presence/absence of LIF, identified in the absence of Dox (FDR<0.05). Middle: Identical representation of the same set of transcripts but measured in the presence of Dox. Right: histogram showing the distribution of LIF-responsive genes across a range of fold changes (X-axis) in the absence (top) and the presence (bottom) of Dox. **(C)** Relative mRNA levels of a set of pluripotency factors normalised to the average expression of all replicates measured in the presence of LIF and the absence of Dox (n=3). **(D)** Validation by RT-qPCR of gene expression genes for a subset of pluripotency factors, presented like in (B). **(E)** Z score violin plots of the four groups of genes identified by RNA-seq in SunTag cells cultured in the absence/presence of LIF/Dox across the conditions indicated on the X-axis. The boxplots are presented as in Supplementary Figure 2D. **(F)** Association of the same four groups with the individual Nanog-binding clusters identified in Figure 3, presented as in Figure 3.

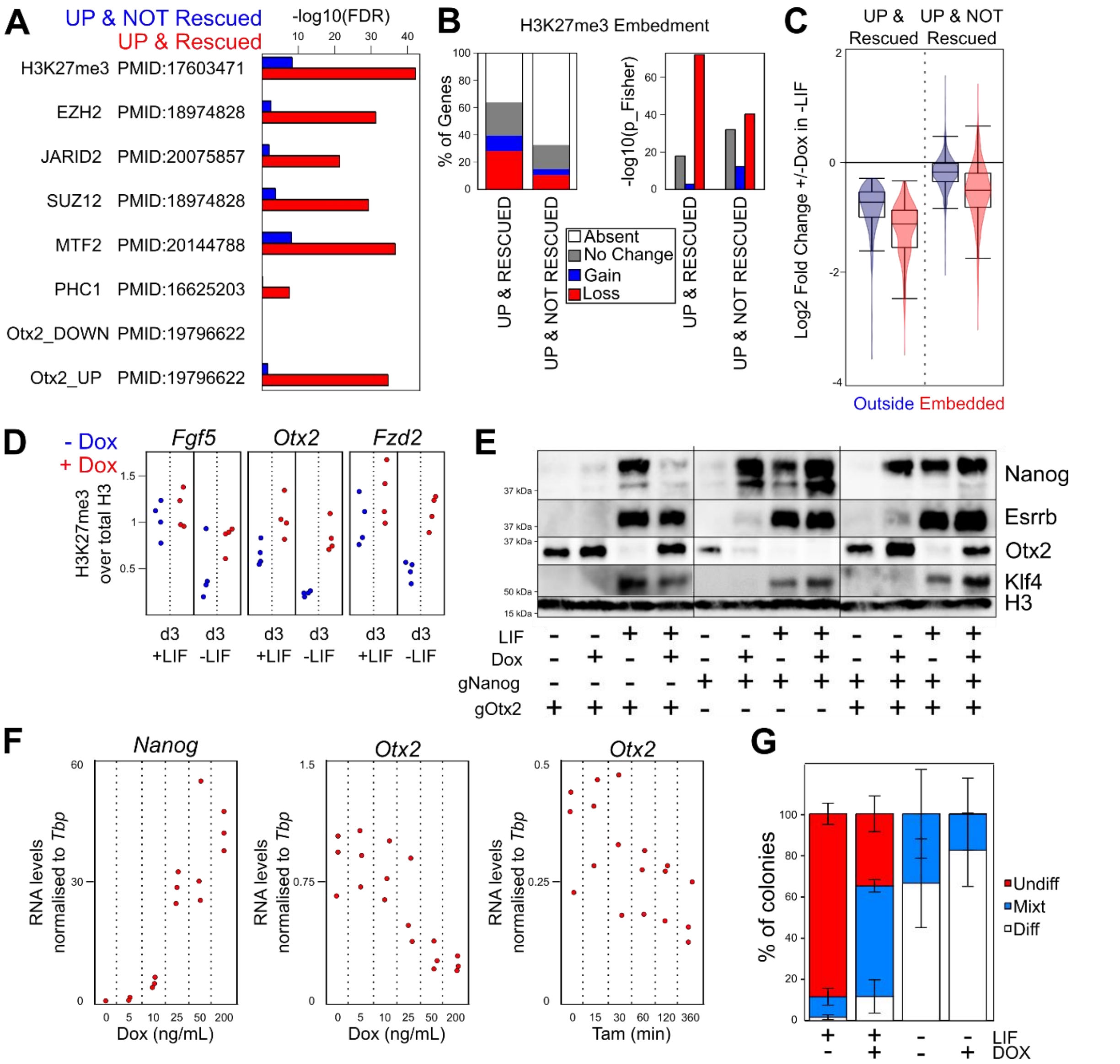

Supplementary Figure 5: H3K27me3 and Otx2 are involved in LIF-independent self-renewal as established by Nanog. (A) Enrichment (-log10_FDR) of UP & NOT rescued and UP & rescued genes for Polycomb Group targets and Otx2 responsive genes. The specific publication used by the Enrichr software (http://amp.pharm.mssm.edu/Enrichr/) to compute the enrichment is shown. **(B)** Proportion (left) and statistical significance (right) of H3K27me3-embedded genes activated without LIF and displaying differential rescue by Nanog (X-axis). **(C)** Violin plot of the log2 fold change of expression for the two previous categories of LIF-responsive genes split in function of their embedment (red) or not (blue) within H3K27me3 domains. The boxplots are presented as in Supplementary Figure 2D. **(D)** Normalised H3K27me3 ChIP-qPCR at three genes showing Nanog-dependent H3K27me3 enrichment in the absence of LIF. **(E)** Western Blot of the indicated factors (right) across multiple conditions (bottom). Note other WB presented in the principal figures correspond exactly to the blots shown here to facilitate direct comparisons between all analysed conditions. **(F)** Normalised mRNA levels of *Nanog* and *Otx2* over a Dox dose-response assay in 44iN and during a time-course of Tamoxifen treatment in Nanog-ERT2 fusion cells (44NERT; RT-qPCR n=3). 44NERT cells are *Nanog* knock-out cells expressing Nanog-ERT2 transgene (Navarro et al., 2012). **(G)** Clonal assay (n=4) in the indicated conditions (X-axis) using SunTag cells that activate *Otx2* exclusively.

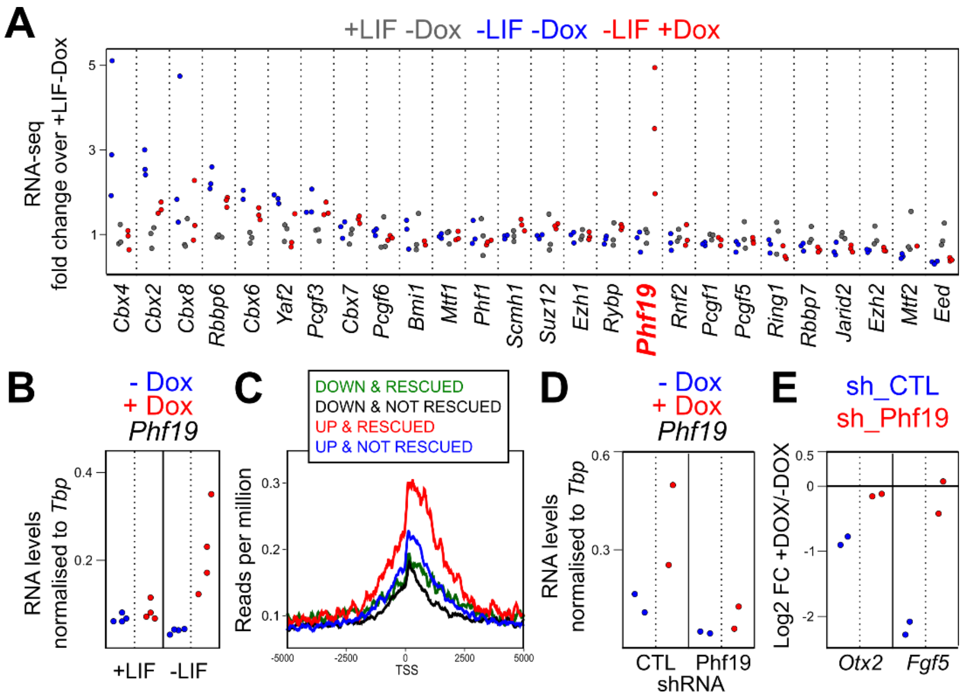

Supplementary Figure 6: Phf19 as a candidate Polycomb protein linking Nanog function to H3K27me3. (A) Analysis of Polycomb group proteins expression in our RNA-seq (n=3) identifies Phf19 as the only member being activated by Nanog in the absence of LIF. **(B)** RT-qPCR validation of *Phf19* induction by Nanog in the absence of LIF (n=4). **(C)** Analysis of Phf19 binding (Ballaré et al., 2012) at the promoter region of genes differentially expressed upon LIF withdrawal and Nanog induction. Note that genes activated during differentiation and downregulated by Nanog (UP & RESCUED, in red), display increased Phf19 enrichment. **(D)** A single clone with effective *Phf19* knock-down (kd) activity was identified upon stable transfection of a shRNA-expressing vector in our Nanog-SunTag cells (clone C1), as compared to cells expressing a scramble shRNA (Ballaré et al., 2012). **(E)** Control and *Phf19* kd Nanog-SunTag cells where cultured in the absence of LIF for 3 days both in the presence and absence of Dox and analysed by RT-qPCR (n=2). The reduction of *Phf19* levels (in red) abolished the ability of Nanog to downregulate *Otx2* and *Fgf5* as observed in control cells (in blue). While these results require a validation through the generation of *Phf19* knock-out cells, they suggest Phf19 contributes to the Nanog-mediated repression of genes that are normally upregulated upon differentiation.
