# Supplementary material for: The molecular logic of Nanog-induced self-renewal in mouse embryonic stem cells
Source: Nat Commun. 2019 Mar 7;10:1109. doi: 10.1038/s41467-019-09041-z (PMC6406003; doi:10.1038/s41467-019-09041-z)
Supplement: Supplementary file 2 — Description of Additional Supplementary Files [file 41467_2019_9041_MOESM2_ESM.pdf]

Description of Additional Supplementary Files

File Name: Supplementary Data 1
Description: Primer and gRNA sequences as well as antibodies information.

File Name: Supplementary Data 2
Description: Global statistics of our sequencing data.
