# Supplementary material for: The molecular logic of Nanog-induced self-renewal in mouse embryonic stem cells
Source: Nat Commun. 2019 Mar 7;10:1109. doi: 10.1038/s41467-019-09041-z (PMC6406003; doi:10.1038/s41467-019-09041-z)
Supplement: Supplementary file 5 — Reporting Summary [file 41467_2019_9041_MOESM5_ESM.pdf]

nature research

Corresponding author(s): Pablo Navarro

Last updated by author(s): Jan 14, 2019

Reporting Summary

Nature Research wishes to improve the reproducibility of the work that we publish. This form provides structure for consistency and transparency in reporting. For further information on Nature Research policies, see Authors & Referees and the Editorial Policy Checklist.

Statistics

For all statistical analyses, confirm that the following items are present in the figure legend, table legend, main text, or Methods section.

n/a	Confirmed	
☐	☒	The exact sample size (*n*) for each experimental group/condition, given as a discrete number and unit of measurement
☐	☒	A statement on whether measurements were taken from distinct samples or whether the same sample was measured repeatedly
☐	☒	The statistical test(s) used AND whether they are one- or two-sided<br>*Only common tests should be described solely by name; describe more complex techniques in the Methods section.*
☐	☒	A description of all covariates tested
☐	☒	A description of any assumptions or corrections, such as tests of normality and adjustment for multiple comparisons
☐	☒	A full description of the statistical parameters including central tendency (e.g. means) or other basic estimates (e.g. regression coefficient) AND variation (e.g. standard deviation) or associated estimates of uncertainty (e.g. confidence intervals)
☐	☒	For null hypothesis testing, the test statistic (e.g. *F*, *t*, *r*) with confidence intervals, effect sizes, degrees of freedom and *P* value noted<br>*Give P values as exact values whenever suitable.*
☒	☐	For Bayesian analysis, information on the choice of priors and Markov chain Monte Carlo settings
☐	☒	For hierarchical and complex designs, identification of the appropriate level for tests and full reporting of outcomes
☒	☐	Estimates of effect sizes (e.g. Cohen's *d*, Pearson's *r*), indicating how they were calculated

Our web collection on statistics for biologists contains articles on many of the points above.

Software and code

Policy information about availability of computer code

Data collection	described thoroughly in the methods
Data analysis	described thoroughly in the methods

For manuscripts utilizing custom algorithms or software that are central to the research but not yet described in published literature, software must be made available to editors/reviewers. We strongly encourage code deposition in a community repository (e.g. GitHub). See the Nature Research guidelines for submitting code & software for further information.

Data

Policy information about availability of data

All manuscripts must include a data availability statement. This statement should provide the following information, where applicable:

- Accession codes, unique identifiers, or web links for publicly available datasets
- A list of figures that have associated raw data
- A description of any restrictions on data availability

All genome-wide datasets are available through the NCBI Gene Expression Omnibus (GEO; https://www.ncbi.nlm.nih.gov/geo), under the accession number GSE118898. The data underlying our ChIP-, ATAC, and RNA-seq analyses are provided as Source Data Files.

Field-specific reporting

Please select the one below that is the best fit for your research. If you are not sure, read the appropriate sections before making your selection.

☒ Life sciences ☐ Behavioural & social sciences ☐ Ecological, evolutionary & environmental sciences

For a reference copy of the document with all sections, see nature.com/documents/nr-reporting-summary-flat.pdf

Life sciences study design

All studies must disclose on these points even when the disclosure is negative.

Sample size	regular number of replicates were performed, as described in the methods and figure captions
Data exclusions	all published data in GEO was used
Replication	all experiments except those related to Phf19 have been replicated in independent biological replicates and different experimental setups. Phf19 experiments are described in detail and included in the paper on the advice of the referees and editor
Randomization	the statistical methods are carefully described in the methods
Blinding	n/a

Reporting for specific materials, systems and methods

We require information from authors about some types of materials, experimental systems and methods used in many studies. Here, indicate whether each material, system or method listed is relevant to your study. If you are not sure if a list item applies to your research, read the appropriate section before selecting a response.

Materials & experimental systems

n/a	Involved in the study	
☐	☒	Antibodies
☐	☒	Eukaryotic cell lines
☒	☐	Palaeontology
☒	☐	Animals and other organisms
☒	☐	Human research participants
☒	☐	Clinical data

Methods

n/a	Involved in the study	
☐	☒	ChIP-seq
☐	☒	Flow cytometry
☒	☐	MRI-based neuroimaging

Antibodies

Antibodies used	described in the methods
Validation	cross-validation with published data; use of gain/loss of function

Eukaryotic cell lines

Policy information about cell lines

Cell line source(s)	E14Tg2a ES cells
Authentication	all mutant cell lines generated for this study have been authenticated by several means (drug resistance, PCR, sequencing) - throroughly described in the methods
Mycoplasma contamination	all cell lines were tested negative
Commonly misidentified lines (See ICLAC register)	n/a

ChIP-seq

Data deposition

☒ Confirm that both raw and final processed data have been deposited in a public database such as GEO.

☒ Confirm that you have deposited or provided access to graph files (e.g. BED files) for the called peaks.

Data access links *May remain private before publication.*	GSE118898
Files in database submission	*Provide a list of all files available in the database submission.*

Genome browser session (e.g. UCSC)	no longer applicable

Methodology

Replicates	described in supplementary material
Sequencing depth	described in supplementary material
Antibodies	described in methods
Peak calling parameters	described in methods
Data quality	described in supplementary material
Software	described in methods

Flow Cytometry

Plots

Confirm that:

☒ The axis labels state the marker and fluorochrome used (e.g. CD4-FITC).

☒ The axis scales are clearly visible. Include numbers along axes only for bottom left plot of group (a 'group' is an analysis of identical markers).

☒ All plots are contour plots with outliers or pseudocolor plots.

☒ A numerical value for number of cells or percentage (with statistics) is provided.

Methodology

Sample preparation	described in methods
Instrument	described in methods
Software	described in methods
Cell population abundance	described in methods
Gating strategy	n/a

☐ Tick this box to confirm that a figure exemplifying the gating strategy is provided in the Supplementary Information.
